# Drug Repurposing Uncovers New Chemical Scaffolds as Potent Urease Inhibitors: A Comprehensive Computational Study

**DOI:** 10.3390/ijms27083561

**Published:** 2026-04-16

**Authors:** Sofía E. Ríos-Rozas, Elizabeth Valdés-Muñoz, Vicente Rojas-Santander, Javier Farías-Abarca, Erix W. Hernández-Rodríguez, Héctor R. Contreras, Jonathan M. Palma, Reynier Suardíaz, Manuel I. Osorio, Osvaldo Yáñez, Luis Morales-Quintana, Daniel Bustos

**Affiliations:** 1 Laboratorio de Bioinformática y Química Computacional, Departamento de Medicina Traslacional, Facultad de Medicina, Universidad Católica del Maule, Talca 3480094, Chile; sofiarios@ug.uchile.cl (S.E.R.-R.); elizabeth.valdes@alu.ucm.cl (E.V.-M.); vicente.rojas.03@alu.ucm.cl (V.R.-S.); javier.farias@alu.ucm.cl (J.F.-A.); ehernandez@ucm.cl (E.W.H.-R.); 2 Basic and Clinical Oncology Department, Faculty of Medicine, University of Chile, Santiago 8350499, Chile; hcontrer@uchile.cl; 3 Doctorado en Biotecnología Traslacional, Facultad de Ciencias Agrarias y Forestales, Universidad Católica del Maule, Talca 3480094, Chile; 4 Center for Cancer Prevention and Control (CECAN), Santiago 8380453, Chile; 5 Facultad de Ingeniería, Universidad de Talca, Curicó 3349001, Maule, Chile; jonathan.palma@utalca.cl; 6 Departamento de Química Física, Facultad de Ciencias Químicas, Universidad Complutense de Madrid, 28040 Madrid, Spain; reysuard@ucm.es; 7 Facultad de Odontología, Universidad Andrés Bello, Santiago Chile, Echaurren 237, Santiago 8370133, Chile; manuel.osorio@unab.cl; 8 Facultad de Medicina, Centro de Investigación Biomédica, Universidad Diego Portales, Ejército 141, Santiago 8370007, Chile; 9 Centro de Modelación Ambiental y Dinámica de Sistemas (CEMADIS), Facultad de Ingeniería y Negocios, Universidad de Las Américas, Santiago 7500975, Chile; oyanez@udla.cl; 10 Multidisciplinary Agroindustry Research Laboratory, Instituto de Ciencias Biomédicas, Facultad de Ciencias de La Salud, Universidad Autónoma de Chile, Cinco Poniente #1670, Talca 7500912, Región del Maule, Chile; luis.morales@uautonoma.cl

**Keywords:** drug-repurposing, virtual screening, consensus docking, metadynamics, urease

## Abstract

*Helicobacter pylori* urease is a key virulence factor and a validated target for anti-infective strategies. In this study, a comprehensive computational workflow was applied to identify potential urease inhibitors through a drug repurposing approach. A curated library was first filtered using permeability-related descriptors and multiparametric scoring. The resulting compounds were evaluated through ensemble and consensus docking across multiple protein conformations and docking engines, followed by XP rescoring, metal–ligand distance analysis, and molecular dynamics simulations. Binding stability and thermodynamic profiles were further assessed using MM-GBSA and well-tempered metadynamics. This integrative strategy led to the identification of several candidate compounds exhibiting favorable docking scores, stable coordination with the catalytic Ni^2+^ center, and consistent binding behavior during molecular dynamics simulations. Notably, selected compounds showed improved relative binding free energy profiles compared to reference inhibitors within the applied computational framework. Overall, this study provides a robust computational pipeline for urease inhibitor identification and highlights repurposed compounds as promising candidates for further experimental validation.

## 1. Introduction

*Helicobacter pylori* (*Hp*) is a microaerophilic Gram-negative bacillus that colonizes the gastric mucosa of approximately 50% of the world’s population, with prevalences exceeding 70% in regions with lower socioeconomic development [1]. Chronic infection is strongly associated with active gastritis, peptic ulcer, mucosa-associated lymphoid tissue (MALT) lymphoma, and gastric adenocarcinoma, the latter being classified by the IARC as a type I carcinogen [2,3]. Despite the availability of combination therapies based on antibiotics and acid suppressants, eradication rates have declined steadily, mainly due to growing antimicrobial resistance to clarithromycin, levofloxacin, and metronidazole. This scenario has prompted the search for new therapeutic strategies, including drug repositioning as an efficient and cost-effective alternative to accelerate the discovery of anti-*Hp* agents [4].

One of the most attractive molecular targets used in multiple studies focused on the development of new therapies is urease, a metalloenzyme essential for the colonization and persistence of *Hp* [5]. Urease hydrolyzes urea into ammonia and carbon dioxide, neutralizing the stomach pH, protecting the microorganism, and allowing it to survive in the acidic environment of the stomach [6]. *Hp* urease (*Hp*U) consists of the UreA and UreB subunits forming a functional unit and assembled into a dodecamer with a binuclear Ni^2+^ active site, coordinated by a highly conserved carbamoylated lysine (KCX) [6,7]. Its indispensable role in bacterial survival and its widespread conservation among different clinically important microorganisms make it an ideal target for inhibitor optimization [8].

Several chemical families of urease inhibitors (UIs) have been reported, encompassing chemically heterogeneous compounds with distinct mechanisms of action and inhibitory activity [9,10]. Classical inhibitors include metal-directed agents such as hydroxamic acids and thiol-containing compounds, represented by acetohydroxamic acid (HAE) [7], β-mercaptoethanol (BME) and SHA/DJM-type derivatives [6], which inhibit urease through coordination of the binuclear Ni^2+^-dependent catalytic center and have been widely used as biochemical and structural references despite their limited clinical applicability. In addition, a variety of synthetic organic inhibitors have been described, including phosphoramidates (NBPT), substituted (di)thioureas, benzothiazoles [11], and quinone-based compounds [12], which generally act through competitive or mixed mechanisms and display inhibitory activities ranging from the micromolar (μM) to the millimolar (mM) scale. Likewise, several natural products, such as flavonoids, alkaloids, and phenolic metabolites, exhibit urease inhibitory activity with moderate potency and high structural diversity [13]. Within this context, coumarins have emerged as a particularly attractive family of UIs, as they combine a versatile chemical scaffold with a well-characterized and tunable pharmacological profile through rational structural substitutions [14,15]. Recent studies indicate that coumarins and their derivatives can act as competitive or mixed urease inhibitors, displaying relevant inhibitory activities in structure–activity relationship evaluations [16]. A critical and often underestimated factor in the design of therapies against Gram-negative bacteria is the intracellular accumulation of compounds, which depends on physicochemical parameters such as polarity, flexibility, lipophilicity, and the presence of primary amines [17,18]. The external structural barriers of Gram-negative bacteria—including the lipopolysaccharide (LPS)-rich outer membrane (OM), low pore permeability, and active efflux systems—limit the effective accumulation of many drugs in the periplasmic and cytoplasmic spaces [19]. In *Hp*, although the architecture of LPS is different from that of classic enterobacteria, it has been shown that the differential accumulation of hydrophobic and charged molecules significantly influences its antimicrobial potency and therapeutic response [20]. Drug repositioning has been enhanced using high-performance computational methodologies that allow extensive libraries of approved compounds or compounds in early clinical stages to be explored [21]. In the case of enzymes of great biomedical relevance, such as *Hp*U, advances in molecular dynamics techniques such as enhanced sampling allow the capture of key conformational transitions, ligand-induced flexibilities, and rare states of the catalytic pocket, which are crucial factors for improving the selection of inhibitor candidates [22]. Among these techniques, metadynamics (MetaD) is an enhanced sampling method used in molecular dynamics to efficiently explore complex free energy landscapes. Its operation is based on the progressive introduction of small bias potentials into the space of collective variables that describe the process of interest. These biases prevent the simulation from remaining trapped in local minimum and push the system toward new configurations. In this way, MetaD allows access to different states or slow transitions that do not emerge in time scales accessible to classical dynamics and, at the same time, makes it possible to reconstruct the free energy profile associated with the phenomenon under study, strengthening the selection of molecules with a higher probability of inhibitory activity in vitro [23]. Additionally, the combined use of consensus docking and ensemble docking has been shown to improve predictive accuracy in virtual campaigns by integrating different docking algorithms and multiple structural conformations of the target protein [24,25]. Ensemble approaches allow the incorporation of the conformational heterogeneity observed experimentally in *Hp*U, while consensus docking reduces dependence on a single scoring function, increasing the robustness of compound prioritization [26,27].

**Figure 1:**
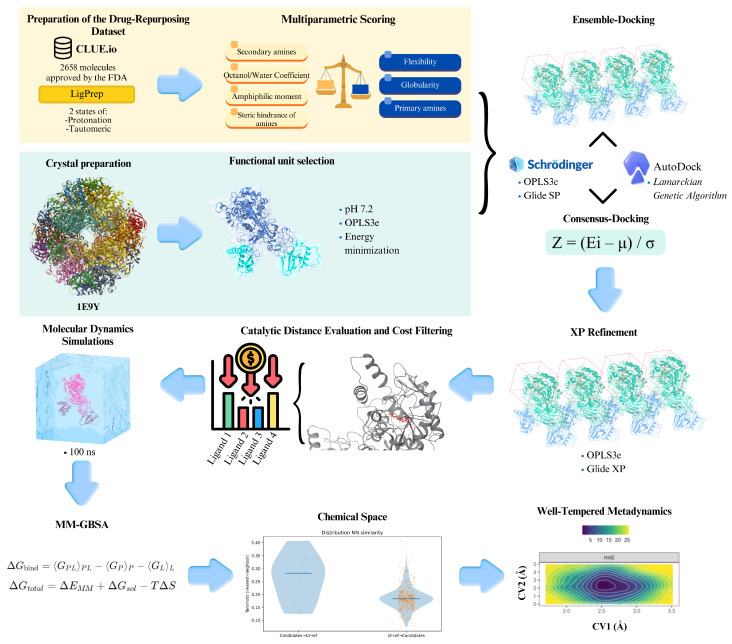
Schematic representation of the integrative computational workflow implemented for the identification of novel *Hp*U inhibitors through drug repurposing. The pipeline begins with the preparation of a curated drug-repurposing dataset obtained from CLUE.io (2658 FDA-approved or late-stage compounds), followed by LigPrep processing to generate relevant protonation and tautomeric states. A multiparametric scoring (MPS) stage incorporating permeability-related descriptors (e.g., flexibility, globularity, amphiphilicity, and amine functionality) was applied to enrich compounds with physicochemical properties compatible with Gram-negative accumulation. In parallel, the *Hp*U crystal structure (PDB ID: 1E9Y) was prepared, and a validated ensemble of receptor conformations was used for ensemble docking with Glide SP (OPLS3e) and AutoDock4. Consensus docking was performed via Z-score normalization and data fusion to reduce engine-specific bias. Top-ranked compounds were subjected to XP docking refinement, followed by catalytic Ni^2+^ distance evaluation and cost-based filtering to prioritize energetically favorable, geometrically plausible, and experimentally accessible candidates. Selected ligands were then evaluated by molecular dynamics simulations (100 ns), MM-GBSA binding free-energy estimation, a chemical space analysis compared to the UI reported in the literature (UI-ref), and finally WT-MetaD to reconstruct two-dimensional free-energy landscapes and rank compounds based on thermodynamic stability within the catalytic pocket. This multistage strategy integrates physicochemical filtering, structural flexibility, energetic refinement, cheminformatics and enhanced sampling to prioritize high-confidence urease inhibitor candidates.

The present study implements an integrative Structure-Based Virtual Screening (SBVS) framework for the rational repurposing of approved and late-stage clinical drugs targeting *Hp*U (Figure 1). The workflow begins with a multiparametric scoring (MPS) stage, in which compounds are prioritized according to Gram-negative accumulation criteria, enriching the library for molecules with a higher probability of intracellular access. The refined subset is subsequently evaluated through Ensemble Docking (ED) across multiple conformational states of *Hp*U to explicitly account for catalytic-site flexibility. To reduce scoring-function bias, docking results from independent engines are integrated using a consensus scoring strategy. Top-ranked candidates are then subjected to molecular dynamics (MD) simulations to assess structural stability and persistence of binding modes under explicit solvent conditions, followed by MM-GBSA binding free-energy estimation. Well-Tempered MetaD (WT-MetaD) is employed to reconstruct free-energy landscapes and quantify thermodynamic stability beyond static docking approximations. In parallel, the structural novelty of the prioritized candidates is evaluated through a bidirectional chemical space analysis against previously reported urease inhibitors curated from public bioactivity databases (UI-ref). By sequentially integrating permeability-aware filtering, conformational ensemble docking, multi-engine consensus scoring, classical MD refinement, and enhanced sampling, this multidimensional pipeline aims to provide a robust and thermodynamically informed prioritization of *Hp*U inhibitor candidates. In this context, the main objective of the present study was to identify and prioritize potential urease inhibitors through an integrative computational pipeline combining permeability-aware filtering, ensemble docking, and thermodynamic evaluation. We hypothesized that compounds capable of establishing stable interactions with the catalytic Ni^2+^ center, while maintaining consistent binding behavior across multiple protein conformations, would represent more reliable inhibitory candidates. Furthermore, the integration of multiparametric filtering with dynamic and thermodynamic analyses was expected to improve the prioritization of compounds with both favorable binding properties and potential biological relevance.

## 2. Results and Discussion

### 2.1. Descriptor Distributions Across the Repurposing Library

To characterize the physicochemical space sampled by the repurposing dataset and evaluate its suitability for Gram-negative permeation, we examined the distribution of seven key descriptors included in the MPS function, together with the frequencies of primary and secondary amine groups (Figure S1A–H). These features were selected based on the permeability rules proposed by Richter et al. [17] and subsequent studies describing the determinants of intracellular accumulation in Gram-negative bacteria. The resulting distributions provide insight into the chemical landscape of the dataset prior to MPS-based refinement and contextualize how far the initial library deviates from, or aligns with, permeation-enabling profiles.

Globularity (Figure S1A) values were generally low (median = 0.103; Q1–Q3 = 0.067–0.154), and the maximum observed value was 1.0. This indicates that most compounds in the repurposing library adopt elongated or moderately disk-like geometries rather than compact spherical shapes. Such low globularity values are favorable for OM transit: non-spherical scaffolds experience fewer steric clashes during porin threading, consistent with permeability models described by Zhao et al. [28], and the shape criteria emphasized by Richter et al. [17], from which the globularity thresholds used in the MPS were derived (≤0.25). The narrow interquartile range further suggests that the dataset is enriched in scaffolds structurally compatible with porin-mediated uptake.

The distribution coefficient (logD, Figure S1B) values showed a median of 1.93 (IQR: 0.26–3.29), spanning a broad chemical space from very hydrophilic (min = −15.23) to lipophilic (max = 12.94) molecules. The central tendency around logD ≈ 2 aligns well with accumulation-competent chemical space described by Richter et al., who observed that moderately polar molecules permeate more effectively than those with excessive hydrophobicity. Extremely high logD values, which hinder OM penetration and increase efflux susceptibility, comprise only a minority of the dataset, consistent with trends in antibacterial collections described by Brown et al. [29]. Thus, the dataset appears well-balanced in terms of lipophilicity for Gram-negative entry.

The molecular weight (MW, Figure S1C) distribution (median = 341 Da; IQR = 259–433 Da) lies well within the structural constraints of general porins, which typically exclude molecules above ~600 Da. Only a few outliers approached the maximal value observed (1695 Da), indicating that most compounds remain within the operational range of passive porin permeation. Although MW alone is a poor predictor of accumulation, as highlighted by Richter et al. [17] and earlier analyses by Brown et al. [29], the predominance of sub-500 Da molecules suggests that steric size is unlikely to be the main limiting factor in this library.

Molecular Planarity (PBF, Figure S1D) values exhibited a median of 0.93 (IQR = 0.76–1.09), reflecting a prevalence of moderately planar scaffolds. Excessive planarity can impair porin permeation due to reduced conformational adaptability, yet some degree of flatness can facilitate target engagement and binding-site complementarity. This distribution agrees with the findings of Kuenemann et al. [30], who demonstrated that planarity and shape metrics modulate ligand–protein interactions and may influence transport pathways. The dataset thus retains a favourable structural balance, avoiding both extreme flatness and excessive 3D complexity.

Rotatable bond (RB, Figure S1E) counts displayed a right-skewed distribution (median = 7; mean ≈ 8.24; max = 44). The majority of compounds fall within the range associated with improved Gram-negative permeation (3–10 RBs), minimizing entropic penalties during porin threading. Flexibility beyond ~10–12 RBs, which has been linked to reduced OM transit efficiency in experimental studies (Brown et al. [29]), is present only in a minority of molecules. These values collectively suggest that the library is structurally predisposed to permeation without being overly rigid or overly flexible.

Steric penalty scores (Figure S1F) were low overall (median = 4; IQR = 2–7; max = 35), indicating that most compounds possess relatively unobstructed shapes with limited bulky substituents. High steric congestion has been identified as one of the strongest negative predictors of OM uptake in MD and porin-permeation models, as highlighted in both Brown et al. [29] and Zhao et al. [31]. The predominance of low-penalty values suggests that steric hindrance is not a major barrier to permeability for most candidates.

Topological Polar Surface Area (tPSA, Figure S1G) values centered near moderate polarity (median = 73.1 Å^2^; IQR = 46–107 Å^2^), which is consistent with ranges tolerated by Gram-negative porins. While classical medicinal chemistry guidelines advocate tPSA < 120 Å^2^ for permeability, Richter et al. [17] demonstrated that distributed polarity, rather than absolute PSA is a more reliable determinant of accumulation. The dataset’s polarity distribution therefore aligns with physicochemical features associated with increased OM penetration and reduced efflux recognition.

Primary and secondary amines (Figure S1H) were present in 11.9% and 12.3% of molecules, respectively. Although these proportions are modest, they are highly relevant: Richter et al. [17] identified the presence of an ionizable nitrogen atom as the single most distinguishing feature of accumulating molecules in Gram-negative bacteria. Such groups adopt protonation states that enhance interactions with porin residues and facilitate periplasmic retention. Consistent with antibacterial chemical space analyses (Brown et al. [29]), the relatively low frequency of amines suggests that only a minority of compounds may readily satisfy the charge-related requirements for accumulation, further justifying their explicit inclusion in the MPS function.

**Figure 2:**
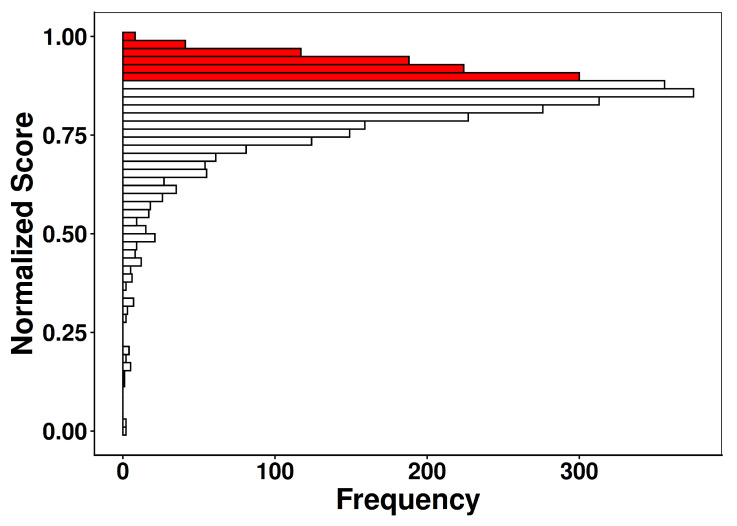
Distribution of normalized MPS across the 3347 compounds in the repurposing library. Bars represent the frequency of compounds at each score interval, with molecules in the top quartile (Q1; score_norm ≥ 0.891) highlighted in red. The MPS displays a right-skewed distribution (median = 0.84; IQR = 0.77–0.89), and 545 molecules (25.0%) fall within the Q1 region. These top-scoring compounds exhibit markedly higher MP-scores (median = 0.92; sd = 0.025), reflecting strong convergence toward physicochemical features associated with Gram-negative permeation.

The normalized MPS (Figure 2) revealed a right-skewed distribution across the 3347 molecules in the repurposing library, with values ranging from 0.00 to 1.00 and a median of 0.84 (IQR = 0.77–0.89). This pattern reflects the intrinsic chemical heterogeneity of the dataset and indicates that, although most compounds exhibit partial alignment with the physicochemical requirements for Gram-negative accumulation, only a smaller subset fully satisfies these criteria. Such enrichment behavior mirrors the trends described by Richter et al. [17] who demonstrated that accumulation-competent molecules tend to occupy a narrow region of chemical space defined by moderate polarity, low globularity, limited steric hindrance, and the presence of ionizable nitrogen atoms. Applying a Q1 cutoff (score_norm ≥ 0.891) identified 545 compounds (25.0%) as top-scoring candidates. Within this subset, MPS were highly concentrated toward the upper range (median = 0.92; mean = 0.93) and exhibited minimal dispersion (S.D. = 0.025), indicating strong convergence toward a shared set of permeation-enabling features. This sharp reduction in variance relative to the full library (S.D. = 0.118) demonstrates that the MPS function effectively prioritizes molecules simultaneously satisfying multiple permeability-related constraints, rather than those meeting only isolated properties.

The resulting distribution highlights two important aspects of library composition and scoring performance. First, although the repurposing dataset spans a broad physicochemical range, a substantial fraction of compounds approaches the permeability-favorable window, consistent with the moderate logD values, low globularity, and restrained steric penalties observed in the descriptor analyses (Figure S1). Second, the Q1-enriched region provides a rational starting point for SBVS: the number of retained molecules (n = 545) is sufficiently large to maintain chemical diversity, yet small enough to increase the efficiency and predictive value of downstream ensemble docking and consensus scoring.

### 2.2. Ensemble and Consensus Docking Analysis

To account for the conformational heterogeneity of *Hp*U and improve the reliability of ligand-ranking, ED was performed using the 25 receptor conformations derived from the crystal structure PDB ID: 1E9Y [7]. The selection of this ensemble was not arbitrary. In our previously published benchmarking study on urease SBVS methodologies [16], the 1E9Y-derived ensemble consistently outperformed those obtained from PDB IDs: 1E9Z, 6QSU, and 6ZJA [6]. Notably, it yielded the highest Pearson and Spearman correlations with experimental IC_50_ values across multiple docking variants. In that same analysis, the 1E9Y ensemble exhibited superior frame-to-frame stability in cavity volume, fewer instances of pose-generation failures, and a more permissive but structurally realistic catalytic architecture, factors that collectively contribute to improved predictive performance.

Consistent with these prior findings, ED applied to the repurposing dataset produced a broader and more informative distribution of docking scores than single-structure docking, reflecting the ability of ligands to sample multiple conformational states of the catalytic pocket. This effect is particularly relevant in urease, where the mobility of flap residues and the dynamic breathing of the metal-containing active site substantially influence ligand accommodation. By interrogating 25 receptor conformations rather than a single static structure, ED reduces pose bias and enriches for binding modes that remain favorable across different structural microstates. Beyond ensemble generation, the aggregation of docking results across conformations was performed through consensus scoring, using Z-score normalization across engines (Glide and Autodock4) followed by minimum-based data fusion. The rationale for this approach is grounded in our prior comparative analysis, where minimum aggregation (“data fusion: minimum”) emerged as the most consistent and discriminative method for recovering experimental activity trends, regardless of scoring term, or pose count. Specifically, the minimum fusion strategy produced the highest correlation values, lowest error metrics (MAE, RMSE), and the most robust inlier ratios across multiple protein structures and docking protocols. This behavior is mechanistically interpretable: the minimum score captures the most favorable receptor–ligand interaction achievable across the conformational ensemble, consistent with the physical expectation that ligands bind preferentially to the subset of states most compatible with their geometry and interaction pattern.

**Figure 3:**
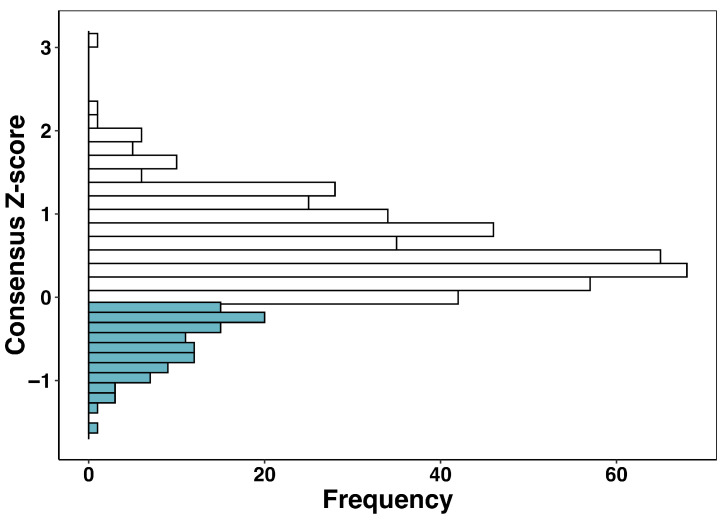
Consensus Z-score distribution obtained from ensemble docking. Histogram of consensus Z-scores computed from ensemble docking across the 25 conformations of *Hp*U derived from the 1E9Y structure. The global distribution (white bars, n = 545) spans a wide range of values (−1.60 to +3.11) with a median of 0.38 (IQR = 0.02–0.81) and a standard deviation of 0.66, reflecting substantial variability in ligand affinity across conformational states. Compounds highlighted in blue (n = 109) correspond to the top 20% scoring ligands according to the minimum-based fusion criterion and exhibit consistently negative consensus Z-scores (−1.60 to −0.11; median = −0.47; S.D. = 0.32). This enriched subset represents ligands with reproducibly favorable interactions across the ensemble, illustrating the discriminatory power of consensus scoring and validating the use of minimum data fusion for prioritizing robust urease binders.

Consensus Z-scores derived from ED displayed in Figure 3 a broad and asymmetric distribution across the 545 compounds retained after MPS refinement. Values ranged from −1.60 to +3.11, with a median of 0.38 (IQR = 0.02–0.81) and a mean of 0.40 ± 0.66. This distribution reflects the intrinsic conformational sensitivity of the urease active site and highlights the stringency of evaluating ligands across 25 receptor conformations. The predominance of positive Z-scores indicates that most ligands achieve only modest affinity relative to the ensemble-wide average, whereas strongly stabilizing interactions are comparatively rare. Application of the minimum-based fusion criterion identified 109 molecules (20% of the dataset) with negative consensus Z-scores (Z ≤ cutoff). This selected subset exhibited an affinity profile markedly distinct from the global population: values ranged from −1.60 to −0.11, with a median of −0.47 and a narrow IQR of −0.71 to −0.27. The internal variance of this group was substantially reduced (S.D. = 0.32) relative to the full dataset (S.D. = 0.66), indicating that high-performing ligands converge toward a consistent and reproducible interaction pattern across receptor conformations. Their consistently negative Z-scores reflect the ability of these ligands to achieve at least one strongly favorable binding pose in the 1E9Y-derived ensemble, a behavior fully aligned with the physical rationale behind minimum data fusion.

These observations reinforce the methodological conclusions established in our prior benchmarking study, where minimum fusion emerged as the most reliable strategy for recovering experimental inhibition trends and minimizing scoring-function noise across different docking engines. Likewise, the enrichment of the left-tail region by a small but coherent set of ligands validates the predictive advantages of ED, which we previously demonstrated to outperform single-structure docking in terms of correlation with experimental IC_50_ values and robustness to conformational artifacts.

### 2.3. Refinement of Ensemble-Selected Ligands by XP Docking, Nickel-Binding Proximity, and Economic Feasibility

**Figure 4:**
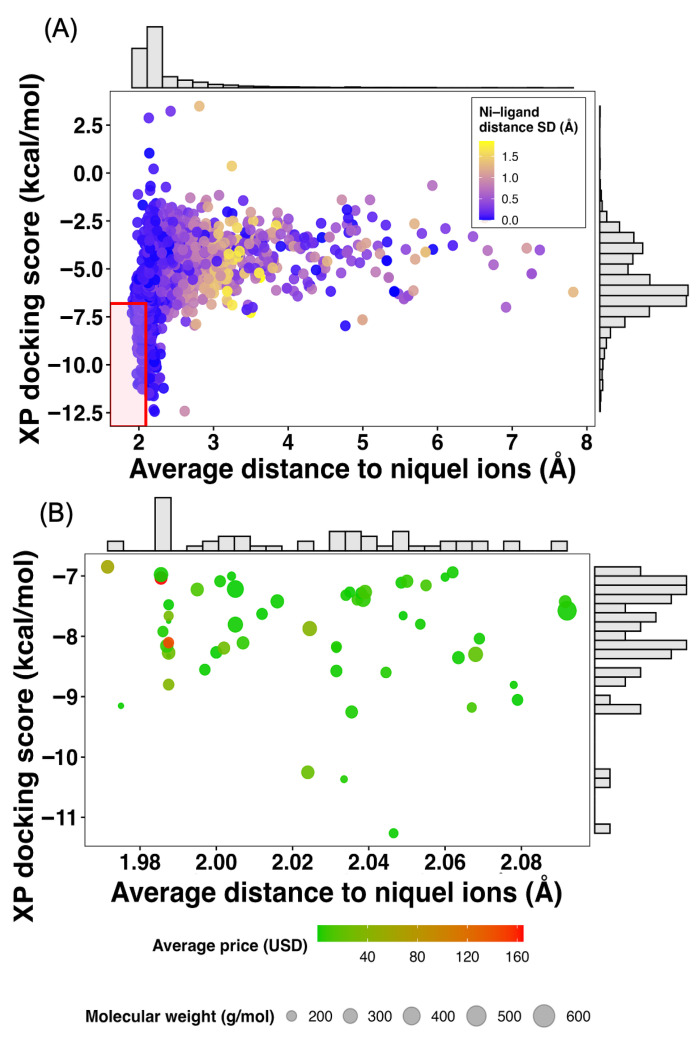
Distance–energy filtering and cost-aware prioritization of urease inhibitors. (**A**) Scatter plot of XP Glide docking score versus the average distance between the ligand center of mass and the two catalytic Ni^2+^ ions for all ligands evaluated at the XP stage. Points are colored according to the standard deviation (SD) of the Ni–ligand distance, reflecting the geometric stability of metal coordination across frames. Marginal histograms show the distributions of docking scores and distances. The red shaded rectangle highlights the physically motivated selection region, defined by simultaneously favorable docking energies (≤−6.805 kcal/mol) and close proximity to the binuclear center (≤2.094 Å), which enriches ligands exhibiting both strong interaction patterns and consistent placement within the catalytic pocket. (**B**) Subset of ligands retained within the selected region, further annotated by average commercial price (color scale) and molecular weight (point size). This representation integrates energetic performance, geometric relevance, economic feasibility, and physicochemical size, enabling a cost-aware prioritization of candidates. Marginal histograms summarize the distributions of docking scores and distances for the selected compounds.

To refine the post–ensemble/consensus shortlist, the selected ligands were re-docked using Glide XP and evaluated jointly by (i) XP docking score and (ii) the average distance between the ligand center-of-mass (COM) and the COM of two catalytic Ni^2+^ ions (Figure 4A). The resulting distribution comprised 2717 docking observations corresponding to 109 unique ligands, reflecting how each ligand behaves across the ensemble conformations.

Across the full dataset, XP scores spanned a broad energetic range (min = −12.4, max = 3.49 kcal/mol), with a median of −6.04 kcal/mol and an interquartile range (IQR) from −6.80 to −4.45 kcal/mol (Table S1). Notably, the global first quartile (Q1 = −6.805 kcal/mol) indicates that only the lower tail of the distribution achieved markedly favorable XP scores, consistent with the expectation that XP refinement increases stringency and reduces the fraction of highly ranked candidates. Importantly, although this threshold is defined from the internal distribution of the dataset, it is not arbitrary in physicochemical terms. In our previous study using the same enzymatic system (*Hp*U), identical reference inhibitors (BME, HAE, and DJM), and comparable Glide XP protocols [10], the most potent control compound (DJM) exhibited an average XP docking energy of −6.119 kcal/mol, with a first quartile value of −6.630 kcal/mol (Table S3). These values define the energetic regime associated with high-affinity binding to the catalytic site. Notably, the threshold applied in the present study (XP ≤ −6.805 kcal/mol) falls within, and is slightly more stringent than this energetic range, effectively aligning the selection criterion with the binding profile of the strongest known inhibitor. This observation highlights that, while absolute docking scores are not directly transferable across independent virtual screening campaigns due to differences in dataset composition and conformational sampling, the energetic window associated with favorable binding remains consistent. Therefore, the use of a data-driven threshold based on the current score distribution, while maintaining consistency with experimentally validated reference compounds, provides a robust and physically meaningful criterion for ligand prioritization.

In parallel, the average Ni^2+^-distance distribution was sharply concentrated near the catalytic region (Q1 = 2.094, median = 2.14 Å; IQR = 2.09–2.40 Å; min = 1.91 Å), while still exhibiting a long right tail (max = 7.82 Å) that captures poses geometrically displaced from the bimetal center (Table S1). Together, these marginal distributions indicate that the dataset contains both (i) poses compatible with close catalytic-site occupancy and (ii) a non-trivial fraction of higher-distance solutions that likely correspond to peripheral or partially solvent-exposed binding modes, which are unlikely to represent catalytically relevant binding poses.

To identify the most catalytically plausible candidates, we defined a joint selection region (shaded box in Figure 4A) requiring XP docking score ≤ −6.805 kcal/mol and average Ni^2+^ distance ≤ 2.094 Å. These thresholds correspond to the Q1 for energy and distance and were chosen to enforce simultaneous energetic favorability and geometric proximity to the dinuclear center, i.e., a conservative proxy for productive engagement of the urease catalytic pocket. Applying this two-dimensional filter retained 277 observations (10.20%) out of 2717 pose–frame instances, corresponding to 55 unique ligands (50.46%) (Table S1). This difference between observation-level and ligand-level retention highlights an important feature of the ensemble: while many ligands can satisfy the joint criteria in at least one conformation, only a smaller subset does so consistently across frames, reinforcing the value of retaining ensemble-derived variability rather than relying on single-structure docking.

Following the physically motivated refinement established in Figure 4A, the subset of ligands occupying the low-energy/short-distance quadrant was further evaluated by integrating chemical feasibility and translational constraints, including compound availability, cost dispersion across vendors, and molecular size. Figure 4B visualizes this multidimensional prioritization by projecting the 55 retained ligands onto the same energetic–geometric space, now colored by average market price and scaled by molecular weight, while Table S2 summarizes the quantitative descriptors underlying the final selection.

Consistent with the enrichment observed in Figure 4A, all ligands shown in Figure 4B reside within a narrow window of XP docking scores (approximately −7 to −11 kcal/mol) and Ni^2+^–ligand distances centered around ~2.0 Å, confirming that energetic favorability and catalytic-site proximity were preserved after collapsing the dataset to unique ligands. Importantly, this projection reveals that strong binding geometry and affinity are not restricted to chemically large or economically prohibitive compounds. Instead, a substantial fraction of ligands with optimal energetic–spatial profiles also exhibit low average prices, broad vendor availability, and moderate molecular weights, reinforcing their suitability for future experimental validation.

The final selection of nine candidate molecules: fenbufen, minoxidil, oxaprozin, fosfosal, ascorbic acid, amfenac, gabapentin, tranexamic acid, and tiludronate was not based on a single threshold but rather on a composite ranking integrating the following criteria: (i) favorable XP docking score, (ii) close average distance to the bimetallic Ni^2+^ center, (iii) low standard deviation of Ni^2+^–ligand distance (indicating geometric stability), (iv) low average market price, (v) low price dispersion across suppliers, (vi) high vendor availability, and (vii) molecular weights generally below ~350 g/mol. This multiparameter strategy ensures that selected ligands are not only computationally promising but also experimentally accessible and chemically tractable. The representative docking poses selected for each of these nine ligands are shown in Figure S2, providing a structural visualization of their binding modes within the catalytic pocket.

Table S2 contextualizes these choices by reporting the 2D structures, energetic and geometric descriptors, pricing statistics, vendor coverage (up to 7 independent distributors), and molecular weights for each selected compound. Notably, most candidates display tight distance distributions (SD ≤ ~0.15 Å) and low-price variability, suggesting reproducible binding modes and practical procurement. The inclusion of chemically diverse scaffolds, ranging from small polar molecules (e.g., ascorbic acid, tranexamic acid) to moderately sized aromatic anti-inflammatory agents (e.g., fenbufen, oxaprozin, amfenac), further supports the robustness of the selection, as it reduces the risk of scaffold-specific bias in subsequent MD and free-energy analyses.

Importantly, this prioritization strategy deliberately extends beyond purely computational scoring. As highlighted by the comparison between Figure 4A,B, ligands with excellent docking scores but high cost, limited availability, large molecular weight, or unstable distance profiles were deprioritized, even if they formally satisfied the energetic–geometric thresholds. This reflects a pragmatic screening philosophy in which biophysical plausibility, chemical stability, and translational feasibility are treated as equally critical dimensions.

### 2.4. Dynamic Stability and Binding Free-Energy for Candidate Selection

To establish an objective criterion for advancing compounds to enhanced sampling simulations, all post-docking MD metrics were explicitly benchmarked against DJM, the most potent inhibitor among the reference controls employed in this study. DJM therefore provides a stringent upper bound for acceptable dynamic stability and binding energetics.

**Figure 5:**
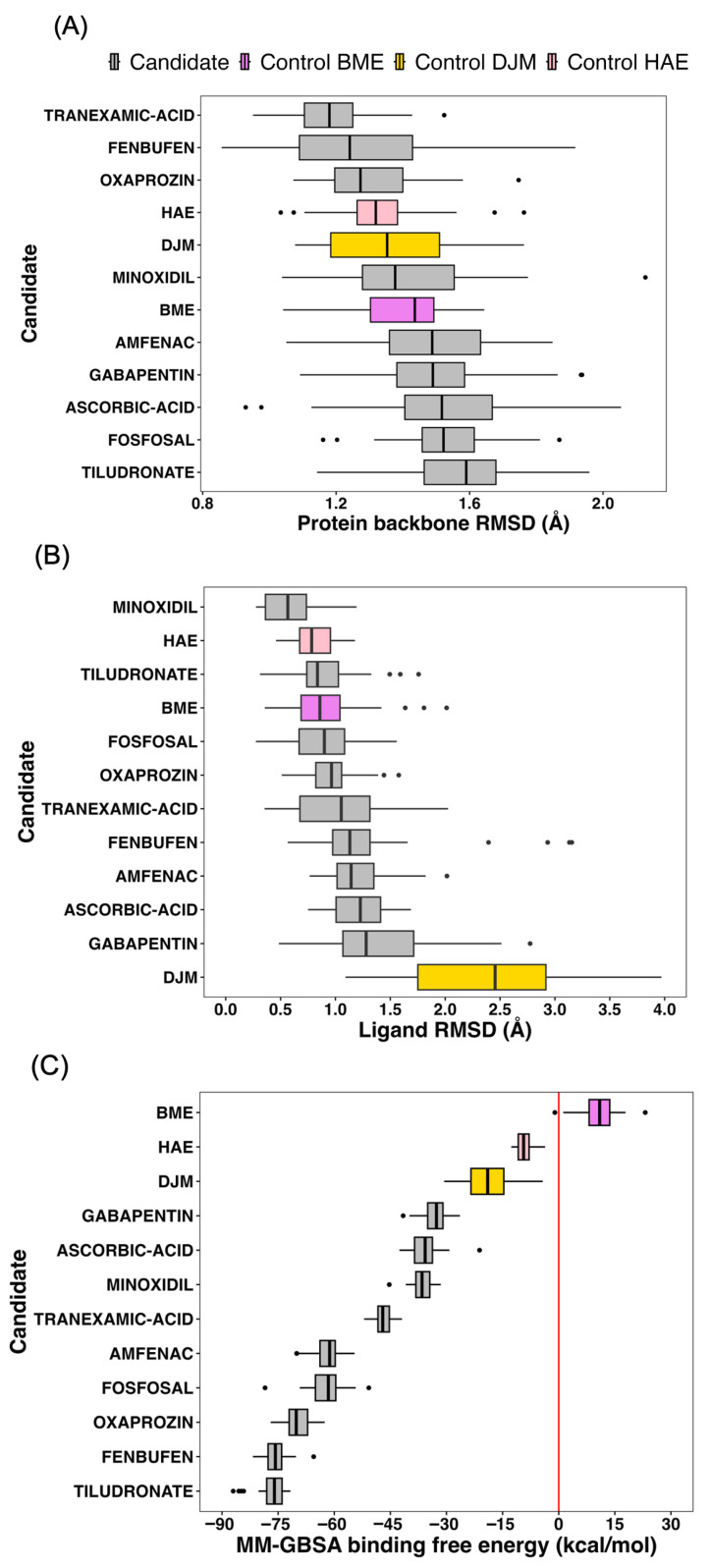
Structural stability and binding energetics of selected urease–ligand complexes during molecular dynamics simulations. Comparative analysis of protein and ligand stability, together with binding free-energy estimates, for the selected candidate compounds and reference controls (DJM, HAE, and BME) based on the last 20 ns of molecular dynamics simulations, corresponding to the fully unrestrained segment following an initial equilibration stage with positional restraints applied to the ligand. (**A**) Distribution of protein backbone RMSD values (Å) for each protein–ligand complex, reflecting the global structural stability of urease upon ligand binding. (**B**) Ligand RMSD distributions (Å) calculated relative to the protein, reporting the positional persistence of each compound within the active site. (**C**) MM-GBSA binding free-energy distributions (kcal/mol) computed over the same time window, with the red vertical line indicating ΔG = 0 kcal/mol. Boxplots represent median values and interquartile ranges, with whiskers extending to 1.5 × IQR and individual points indicating outliers.

Analysis of protein backbone RMSD profiles (Figure 5A; Table S3) shows that all selected candidates maintain backbone deviations comparable to, or lower than, those observed for DJM. While DJM induces moderate backbone fluctuations consistent with its strong interaction with the catalytic site, none of the candidate complexes exhibit destabilization beyond this reference envelope. Median backbone RMSD values for candidates remain within a narrow range and overlap with the DJM distribution, indicating that candidate binding does not compromise the global structural integrity of urease. Ligand RMSD analysis (Figure 5B; Table S4) provides a more discriminating metric. DJM displays the largest positional fluctuations among all systems, with a broad RMSD distribution extending above 3 Å, reflecting its known dynamic binding mode within the active site. In contrast, the majority of candidate ligands exhibit significantly lower ligand RMSD medians and interquartile ranges, indicating tighter positional confinement throughout the final 20 ns of unrestrained MD. Candidates such as fenbufen, oxaprozin, fosfosal, minoxidil, tranexamic acid, and tiludronate consistently show ligand RMSD distributions well below those of DJM, demonstrating superior anchoring within the catalytic pocket. This reduced positional variability suggests a lower entropic penalty upon binding and supports the presence of stable, well-defined interaction networks around the Ni^2+^ center.

MM-GBSA binding free-energy calculations (Figure 5C; Table S5) further refine the comparison. DJM exhibits a strong and favorable binding free-energy distribution, serving as the energetic reference. Notably, all candidates reach MM-GBSA medians that surpass DJM, while maintaining substantially lower variance. In particular, tiludronate, fenbufen, oxaprozin, fosfosal, and amfenac show consistently favorable MM-GBSA profiles with narrow distributions, indicating reproducible energetic stabilization across the ensemble of frames. In contrast, candidates displaying either weaker average MM-GBSA values or broader distributions, despite acceptable RMSD behavior were deprioritized, as energetic inconsistency suggests sensitivity to local conformational fluctuations.

Taken together, DJM defines a conservative and mechanistically meaningful reference across the three post-docking dimensions evaluated: protein backbone stability, ligand positional persistence, and binding free energy. In terms of ligand RMSD and MM-GBSA binding free energy, all candidate compounds display more favorable and more stable profiles than DJM, exhibiting lower positional fluctuations and consistently stronger or comparable binding free energies throughout the final 20 ns of unrestrained MD simulations. With respect to protein backbone RMSD, not all candidates stabilize the protein to the same extent as DJM. However, the maximum difference observed between DJM and the least stabilizing candidate (tiludronate) remains below 0.3 Å, a magnitude that is well within the range generally considered indicative of a structurally stable protein in long-timescale MD simulations. Importantly, none of the candidate complexes exhibit signs of abnormal backbone destabilization or global conformational drift relative to the DJM-bound reference. Overall, these results demonstrate that all candidates evaluated at this stage satisfy the dynamic stability and energetic criteria required to advance to enhanced sampling simulations. Consequently, fenbufen, minoxidil, oxaprozin, fosfosal, ascorbic acid, amfenac, gabapentin, tranexamic acid, and tiludronate were all selected for subsequent analysis.

### 2.5. Chemical Space Novelty Assessment Relative to Reported Urease Inhibitors

To evaluate the structural novelty of the nine repurposed candidates relative to previously reported urease inhibitors (UI-ref), a bidirectional nearest-neighbor (NN) similarity analysis was performed using ECFP4 fingerprints and the Tanimoto coefficient (Figure S3). In the Candidates → UI-ref direction (n = 9), the NN similarity distribution exhibits a median of 0.282 (p10 = 0.162; p90 = 0.354). This indicates that, for each candidate, the closest previously UI-ref remains only moderately similar at best. Importantly, no candidate displayed a NN similarity ≥ 0.5, and none approached higher similarity thresholds (≥0.7 or ≥0.85), which are commonly associated with scaffold-level relatedness or close analog relationships. In the reciprocal UI-ref → Candidates direction (n = 338), the similarity distribution shifts toward even lower values (median = 0.183; p10 = 0.138; p90 = 0.238). This suggests that, for the majority of known UI-ref, the most similar molecule within the candidate set remains structurally distant. In other words, the repurposed candidates do not densely cover the previously explored UI-ref chemical space. The absence of NN similarities above 0.5 in both directions supports the conclusion that the selected candidates do not represent close analogs of UI-ref under conventional fingerprint-based similarity metrics. Instead, they appear to occupy peripheral or previously underexplored regions of the UI-ref chemical landscape. These results indicate limited chemical space overlap between the repurposed candidates and the UI-ref dataset, supporting the structural novelty of the identified scaffolds. This observation strengthens the repositioning rationale of the present study, as the computational workflow did not merely rediscover known chemotypes but rather prioritized chemically distinct molecules that converge toward favorable thermodynamic binding behavior.

Beyond the global distribution of compounds in chemical space, our analysis highlights the enrichment of specific chemotypes that may be particularly relevant for urease inhibition. Notably, heteroaromatic scaffolds were consistently represented among the top-ranked candidates, suggesting their role in stabilizing interactions within the catalytic environment. In addition, the presence of aromatic amine groups was recurrent, which may facilitate hydrogen bonding and electrostatic interactions with key residues surrounding the Ni^2+^ center. Furthermore, cyclic amine motifs, particularly piperidine-like structures, were frequently observed among high-ranking compounds. These features may contribute to conformational adaptability and optimal positioning within the active site. Together, these chemotypes define a structural framework that is compatible with both catalytic-site proximity and favorable energetic profiles. Importantly, these findings provide actionable guidance for future optimization strategies, indicating that the incorporation or preservation of these functional groups could enhance binding affinity and catalytic-site engagement in next-generation UI.

### 2.6. WT-MetaD–Based Free Energy Analysis of Selected Urease Inhibitor Candidates

WT-MetaD simulations were performed to refine the thermodynamic ranking of the nine selected candidate inhibitors relative to the three reference controls (DJM, HAE, and BME). The analysis focused on the reconstruction of free-energy landscapes along two mechanistically meaningful collective variables (CVs) describing ligand positioning within the urease catalytic site (see Section 3.8). For each ligand, the free-energy span (ΔF) was calculated independently for CV1 and CV2 as the difference between the maximum and minimum free-energy values sampled, and the mean of both contributions was used as an integrated ranking metric (Table 1). Within the reference set, DJM exhibited a lower mean (ΔF) than HAE and BME, consistent with its experimentally established higher inhibitory potency [6] and its role as the most stringent benchmark in this study. HAE displayed intermediate ΔF values, whereas BME showed the largest free-energy spans, reflecting a shallower and more dispersed thermodynamic profile. This internal ordering (DJM ≫ HAE ≈ BME) provided a coherent reference framework against which candidate ligands could be evaluated. Comparison against DJM revealed that ascorbic acid, fosfosal, fenbufen, and oxaprozin exhibit mean (ΔF) values lower than that of DJM, indicating a more confined and energetically favorable free-energy landscape across both CVs. These compounds therefore surpass DJM under the WT-MetaD criterion, suggesting enhanced thermodynamic stability within the catalytic pocket. Importantly, all remaining candidates display mean (ΔF) values lower than those of HAE and BME, positioning them consistently above the weaker reference inhibitors.

**Figure 6:**
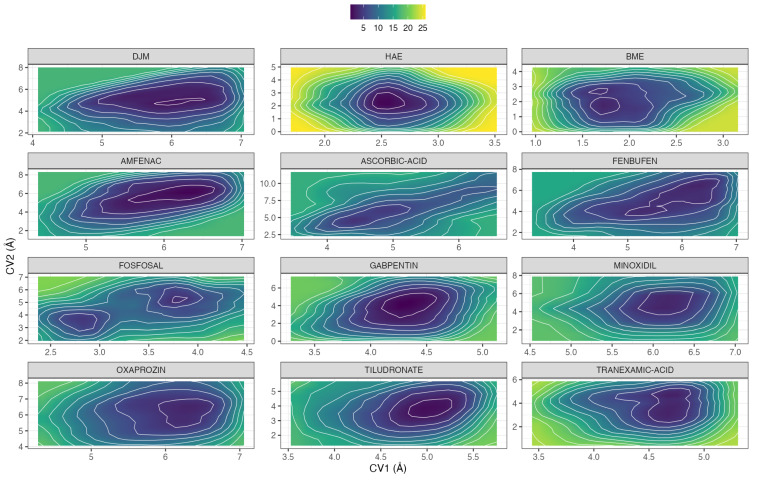
Two-dimensional free-energy landscapes obtained from well-tempered meta-dynamics simulations. Two-dimensional free-energy surfaces (FES) reconstructed from WT-MetaD simulations for the selected candidate ligands and reference controls (DJM, HAE, and BME). Free-energy maps are projected onto the two collective variables (CV1 and CV2), which describe ligand–active-site degrees of freedom relevant to coordination within the urease catalytic pocket. The color scale represents the free-energy value (ΔF, kcal/mol), with darker regions indicating low-energy, thermodynamically favorable basins and lighter regions corresponding to higher-energy states. Contour lines denote iso-free-energy levels, highlighting the shape, depth, and continuity of the sampled minima. Differences in basin localization, compactness, and energy dispersion across ligands reflect distinct binding stability and conformational preferences within the bimetallic Ni^2+^ center, providing a comparative thermodynamic framework for ranking candidates relative to the experimental reference inhibitors. CV1 corresponds to the distance between the center of mass (COM) of ligand heavy atoms and the Ni^2+^ cluster and CV2 is computed from the distance between the COM of the ligand and the COM of the key catalytic residues KCX219, H274, C321, D362, and A365.

The two-dimensional free-energy surfaces (Figure 6) provide a structural and thermodynamic context to the numerical ranking derived from WT-MetaD (Table 1), revealing ligand-specific patterns of confinement, heterogeneity, and accessibility within the urease active site. The reference inhibitor DJM displays a dominant low-energy basin centered near the bimetallic Ni^2+^ cluster, consistent with its known high inhibitory potency. However, this basin extends over a relatively broad region along both CVs, indicating that while DJM remains tightly bound, it retains a measurable degree of positional flexibility within the catalytic pocket. Among the candidate compounds, distinct binding behaviors emerge rather than a single uniform pattern. Fosfosal, in particular, exhibits two clearly separated low-energy minima, suggesting the coexistence of alternative metastable binding modes within the active site. This multimodal landscape is indicative of conformational adaptability rather than simple confinement, yet both minima remain thermodynamically favorable and spatially proximal to the catalytic region. In contrast, compounds such as oxaprozin, ascorbic acid, tiludronate, and minoxidil present single dominant basins with moderate dispersion, reflecting stable binding configurations without excessive fragmentation of the free-energy surface. Fenbufen, while ranking favorably in terms of ΔF, displays an elongated basin rather than a sharply localized minimum, pointing to persistent low-energy mobility along one CV rather than strict geometric locking.

A clear distinction is observed for the weaker reference controls HAE and BME. These ligands uniquely access the lowest ranges of both CVs, reaching values of approximately 3.5 Å in CV1 and ~5 Å in CV2, ranges that are not sampled by the majority of candidate compounds (with fosfosal and tiludronate being partial exceptions). This extended exploration toward shorter distances reflects greater positional freedom and reduced confinement, which manifests as broader, shallower basins and higher free-energy penalties upon displacement from the preferred binding region. Together, these features highlight that WT-MetaD discriminates ligands not only by basin depth (ΔF) but also by landscape topology, including basin multiplicity, elongation, and CV accessibility. Importantly, candidate ligands outperform DJM in terms of ΔF ranking while maintaining binding modes that are thermodynamically stable and spatially compatible with the catalytic Ni^2+^ center, despite not necessarily exhibiting more compact basins. This nuanced interpretation underscores the value of combining numerical free-energy metrics with qualitative FES inspection when prioritizing ligands for subsequent enhanced-sampling and mechanistic analyses.

The one-dimensional free-energy projections along CV1 and CV2 (Figures S4 and S5) reinforce the conclusions drawn from the two-dimensional maps. Ligands outperforming DJM typically display narrow and deep minima along both CVs, reflecting persistent proximity to the Ni^2+^ cluster (CV1) and stable confinement within the catalytic residue environment (CV2). In contrast, DJM, while clearly superior to HAE and BME, retains broader minima, consistent with its intermediate ΔF values relative to the top-ranked candidates.

Taken together, the WT-MetaD results demonstrate that the selected candidates not only meet but, in several cases, exceed the thermodynamic performance of the most potent reference inhibitor DJM. Specifically, ascorbic acid, fosfosal, fenbufen, and oxaprozin emerge as top-ranked compounds with more favorable free-energy confinement than DJM, while the remaining candidates consistently outperform HAE and BME. This outcome is fully consistent with prior WT-MetaD analyses reported by Ríos-Rozas et al. [10] where reductions in ΔF and increased basin compactness correlated with enhanced inhibitory potential.

### 2.7. Repurposing Relevance of Selected Urease Inhibitor Candidates

Among the four top-ranked candidates, ascorbic acid is the only compound for which indirect effects on urease activity have been historically reported. Early biochemical studies demonstrated that ascorbic acid could inhibit urease only under non-physiological conditions, requiring the presence of redox-active metal ions such as Cu^2+^ or Fe^3+^, where inhibition arises from metal reduction and reactive oxygen species generation rather than from direct interaction with the urease Ni^2+^ catalytic center [32,33]. Importantly, no IC_50_, K_i_, or structure-based evidence supports ascorbic acid as a bona fide urease inhibitor acting on the native bimetallic active site, and no modern repurposing studies have positioned it as a urease-targeted agent. For fosfosal (fosfosalicylic acid), fenbufen, and oxaprozin, the literature is even more limited. No reports describe these molecules as urease inhibitors, bacterial enzyme modulators, or anti-virulence agents, either experimentally or in silico [34,35,36,37,38]. Notably, fenbufen has been reported to bind bacterial dihydrofolate reductase in unrelated contexts, but without quantitative potency or relevance to urease biology [38,39]. Oxaprozin and fosfosal have not been evaluated against urease or other Ni^2+^-dependent enzymes, and available studies focus exclusively on their anti-inflammatory or analgesic properties [34,35,36,40]. Collectively, these observations confirm that the identification of these compounds as thermodynamically favorable urease binders represents a previously unexplored chemical–biological association.

Despite the lack of precedent in urease inhibition, all prioritized compounds possess well-characterized clinical or preclinical profiles, which is a central advantage of the repurposing approach [41]. Ascorbic acid is a widely used nutritional supplement with an excellent safety profile at physiological doses and extensive clinical exposure. Beyond its antioxidant role, it has demonstrated adjunct antibacterial and anti-virulence effects, including inhibition of biofilm formation and synergistic activity with conventional antibiotics against multiple Gram-negative pathogens [42,43]. These properties, while not urease-specific, support its candidacy as a host-compatible modulator in antimicrobial strategies.

Fosfosal, fenbufen, and oxaprozin are non-steroidal anti-inflammatory drugs (NSAIDs) or NSAID-related agents with established pharmacokinetics, oral bioavailability, and known dosing regimens [44,45,46,47,48]. Oxaprozin, in particular, exhibits high oral bioavailability and a long plasma half-life, which could be advantageous for sustained target engagement [49,50]. Fenbufen, although withdrawn in some markets due to rare hepatotoxicity, remains pharmacologically well understood and has served as a scaffold in mechanistic studies [51]. Fosfosal, a phosphate prodrug of salicylic acid prodrug, shares the extensive clinical history of salicylates, with predictable metabolism and safety considerations [52].

### 2.8. Broader Implications for Homologous Ureases

Urease is not exclusive to *Hp* but is widely distributed among a variety of clinically relevant pathogens, including *Proteus mirabilis*, *Klebsiella pneumoniae*, and *Staphylococcus saprophyticus*, where it plays a central role in virulence through pH modulation and host colonization. Importantly, ureases across different species share a highly conserved catalytic architecture centered on a binuclear Ni^2+^ active site, which is essential for enzymatic activity [53,54,55]. This Ni-dependent catalytic mechanism, involving urea hydrolysis via a metal-activated nucleophile, is strongly conserved across organisms [56].

In addition to mechanistic conservation, previous studies have demonstrated structural similarities between ureases from different species that enable cross-species modeling and inhibitor design. For example, homology modeling of *Hp*U based on the structure of *Klebsiella aerogenes* has been successfully used to investigate inhibitor binding, supporting the conservation of the active-site geometry [57]. Given this structural and mechanistic conservation, the interaction patterns identified in this study, particularly those involving coordination with the Ni^2+^ cluster and stabilization of the active-site environment, may be transferable to homologous ureases. This suggests that the prioritized compounds could serve as potential starting points for the development of broad-spectrum urease inhibitors. However, differences in surrounding residues, access channels, and flap dynamics may influence ligand binding and specificity across species. Therefore, further studies, including cross-species docking, MD simulations, and experimental validation, will be necessary to assess the generalizability of these findings.

## 3. Materials and Methods

### 3.1. Preparation of the Drug-Repurposing Dataset

The CLUE.io database (https://clue.io/repurposing) contains curated records of compounds that are either FDA-approved or in advanced clinical development (accessed on 5 September 2025). A total of 2658 molecules were downloaded in “.SMILES” format and converted to “.sdf”. Only compounds classified as FDA-approved or in Phase III of traditional drug-development pipelines were retained for this study. All molecules were subsequently prepared in Maestro using LigPrep (Schrödinger Release 2023-3) [58], setting the protonation environment to pH 7.2 to approximate physiological conditions and ensure realistic ligand ionization states during preparation. Although urease remains catalytically active over a broad pH range, near-neutral conditions are commonly used in structure-based drug design workflows to model biologically relevant protonation equilibria. For each molecule, up to two tautomeric states and two protonation states were generated using Epik (Schrödinger Release 2023-3) [59], ensuring the inclusion of relevant ionization microstates under physiological conditions.

### 3.2. Multiparametric Scoring and Preliminary Prioritization

To enrich early candidate selection with parameters linked to small-molecule permeation and retention in Gram-negative bacteria, each compound from the CLUE.io database in the repurposing library was evaluated using a multiparametric scoring function inspired by the accumulation rules described by Richter et al. [17]. The scoring framework integrates physicochemical and geometric descriptors that have been experimentally correlated with antibiotic accumulation in *Escherichia coli* and other Gram-negative species.

The following descriptors were computed for every compound in the library:

- Molecular weight (MW);
- Topological polar surface area (tPSA);
- LogD_7.4_ (and LogD at pH 7.4);
- Number of rotatable bonds (RB);
- Net charge at physiological pH (determined with Epik at pH 7.0);
- Hydrogen-bond donors and acceptors (HBD/HBA);
- Presence and type of amine functionality (primary, secondary, tertiary);
- Globularity;
- Amphiphilic moment (vsurf_A);
- aromatic fraction.

These variables include the most predictive signatures of Gram-negative accumulation, namely: the presence of a non-sterically hindered primary amine, low globularity (≤0.25), restricted flexibility (≤5 rotatable bonds), and a moderate amphiphilic moment that enhances porin translocation.

To integrate these descriptors into a single prioritization metric, each property was normalized to a 0–1 scale and weighted according to its reported contribution to membrane penetration and accumulation. Specifically, sterically accessible amines, low RB counts, and low globularity were assigned higher weights, reflecting their dominant role in the ability of a ligand to traverse Gram-negative porins and avoid efflux. Conversely, properties such as excessive polarity (tPSA > 120 Å^2^) or high MW (>600 Da) were penalized based on prior accumulation studies.

A final MPS was computed as the weighted sum of all normalized descriptors:

$$MPS=\;\sum_{i=1}^{n}w_{i}\;\cdot \;d_{i}$$

where $d_{i}$ represents the normalized descriptor value and $w_{i}$ the empirical weight derived from the accumulation rules described in Richter et al. [17] and adapted to the urease-focused chemical space of this study.

Finally, compounds were filtered based on an optimal physicochemical window, retaining only those satisfying the following thresholds:

- RB ≤ 5;
- Globularity ≤ 0.25;
- Primary amine present and non-hindered;
- MW ≤ 600 Da;
- tPSA ≤ 120 Å^2^;
- Net charge between 0 and +1 at pH 7;
- 0 < LogP < 4, avoiding both excessive hydrophobicity and poor solubility.

Compounds were then ranked according to their final MPS, and only those in the top quartile (best 25%) of the score distribution were retained for subsequent SBVS. This multiparametric refinement ensures that molecules entering the docking stages are not only chemically diverse and drug-like but also predisposed to accumulate in Gram-negative bacteria, a key requirement for the development of effective UI.

### 3.3. Ensemble Docking Against Multiple HpU Conformations

To account for the conformational flexibility of *Hp*U and to improve docking reliability, an ED strategy was implemented [60]. The receptor conformations used in this study were not generated de novo, but instead obtained from a previously published work by our group [16]. In that study, four experimentally resolved structures of *Hp*U (PDB IDs: 6ZJA, 6QSU, 1E9Y, and 1E9Z) were subjected to 2 μs all-atom MD simulations, from which 25 representative frames per structure were extracted using a Bio3D clustering protocol [61]. All MD simulations were performed using a single αβ heterodimeric unit of *Hp*U, which contains the complete catalytic environment, including the binuclear Ni^2+^ center and all key residues involved in substrate binding and catalysis. A comprehensive benchmarking analysis revealed that the ensemble derived from PDB ID: 1E9Y consistently achieved the highest correlation with experimental IC_50_ values across multiple SBVS methodologies, outperforming ensembles obtained from the other three structures. Furthermore, ED was identified as one of the best-performing strategies for recovering experimental inhibition trends, particularly when combined with appropriate scoring functions and data-fusion schemes. On this basis, the present work adopts the validated set of 25 conformational states derived from 1E9Y, taking advantage of their previously demonstrated predictive value and ensuring methodological continuity with our published framework.

Although the receptor conformations were reused from prior work, all docking grids were generated anew for this study to ensure consistency between Glide [62] and AutoDock4 [63]. For Glide, each of the 25 receptor frames was imported into Maestro and prepared with the Protein Preparation Wizard using the OPLS3e force field [64], assigning protonation states at pH 7.2, the optimal activity range for *Hp*U. The docking grid was centered using the centroid defined by nine key catalytic residues: His136, His138, KCX219, His221, His248, His274, Cys322, Arg338, and Asp362, together with the binuclear Ni^2+^ center. Two nested cubic grids were constructed to adequately sample the catalytic cavity: an inner grid of 15 Å × 15 Å × 15 Å to restrict ligand placement within the active site, and an outer grid of 36 Å × 36 Å × 36 Å defining the receptor box. No metal-coordination restraints were applied, consistent with our previous benchmarking results indicating that unrestrained docking better preserves methodological neutrality when screening chemically diverse or previously uncharacterized ligands, such as repurposed drugs.

For AutoDock4, grids were likewise generated for the same 25 conformations. Protein and ligand structures were converted to PDBQT format using AutoDockTools 1.5.7, and a cubic search box of 24 Å per side was centered on the same catalytic centroid used for Glide. Docking calculations were performed with an exhaustiveness value of 16. The Ni^2+^ ions were retained without additional parameterization, relying on Autodock4′s scoring function to penalize unrealistic geometries without the need for explicit metal restraints.

Docking calculations were performed across all 25 conformational frames for both docking engines. For each receptor conformation, Glide-SP and AutoDock4 generated up to 10 binding poses per ligand. Consequently, each ligand underwent a maximum of 250 independent docking evaluations (25 receptor conformations × 2 docking engines × up to 10 poses per engine). This strategy produced a structurally diverse ensemble of receptor–ligand interaction geometries, explicitly accounting for catalytic-site conformational heterogeneity and scoring-function variability. The use of ED to incorporate receptor flexibility has been shown to significantly improve pose prediction accuracy and VS enrichment compared to single-structure approaches [21,24,65]. In parallel, consensus strategies combining multiple docking engines have been widely demonstrated to reduce scoring bias and improve robustness and reproducibility in ligand ranking [66]. Moreover, the integration of ED with consensus scoring has been reported to further enhance screening performance by capturing both structural variability and scoring-function complementarity [67,68]. By expanding the sampling across receptor states and independent scoring algorithms, this design reduces the risk of conformation-specific or engine-specific bias and enables robust downstream consensus scoring and pose prioritization.

### 3.4. Consensus Docking Strategy

To integrate the results obtained with Glide and AutoDock4 and reduce the methodological biases intrinsic to each docking engine, a consensus docking strategy was implemented. All docking outputs generated from the 25 conformational frames and both programs were merged into a unified dataset containing the fields: Ligand, Frame, Energy, Pose, and Program. Because Glide and AutoDock4 produce scores on different numerical scales, a Z-score normalization was applied to each program independently, allowing the energies to be expressed in a comparable statistical space. The Z-score for each docking energy $E_{i}$ was computed as:

$$Z_{i}=-\left(\frac{E_{i}-\mu }{\sigma }\right)$$

where *μ* and *σ* correspond to the mean and standard deviation of all docking energies within the same program. The negative sign was introduced to ensure that more favorable (i.e., lower) docking energies yield higher Z-score values, thereby preserving the correct energetic ranking of ligands across different docking engines. For each ligand, the final consensus score (Consensus_Z) was calculated as the average of its normalized Z-scores across both docking engines, thereby integrating Glide and AutoDock4 into a single comparative metric. This averaging was performed using the normalized Z-scores obtained independently from each docking engine, ensuring equal contribution of Glide and AutoDock4 to the final ranking. This approach minimizes program-specific scoring biases and is consistent with widely used consensus-docking methodologies reported in the literature [25,66,67,69,70,71].

In addition to Z-score integration, a data-fusion step was incorporated to capture the most favorable interaction observed across the conformational ensemble. Based on our previously published benchmarking study [16], in which six data-fusion schemes were systematically evaluated, the minimum-energy fusion method was identified as the strategy that most consistently reproduced experimental IC_50_ values, outperforming approaches based on medians or means [16].

Once the consensus scores were calculated, descriptive statistics (mean, standard deviation, quartiles) were computed. Ligands with the highest Consensus_Z values (top 20%) were selected for refinement. This threshold captures the top 20% of molecules with the most favorable energetics, yielding a subset that is both chemically diverse and consistently supported across docking engines and receptor conformations.

### 3.5. XP Refinement, Catalytic Distance Evaluation, and Cost Filtering

Following ED and consensus docking, the top-ranked ligands were subjected to an additional refinement stage using Glide XP (Extra Precision, [72]), with the aim of improving discrimination between true binders and false positives and achieving a more accurate description of ligand positioning within the catalytic cavity. XP docking was conducted using the same 25 receptor grids employed for the SP ensemble-docking stage, thus ensuring full comparability between precision levels and avoiding grid-dependent artifacts. For each ligand, up to 10 poses per conformational frame were generated under the OPLS3e force field, maintaining the grid centered on the binuclear Ni^2+^ active site.

Because urease inhibition is strongly associated with the chelating capacity of ligands toward the Ni^2+^ cluster, a distance-based evaluation was incorporated. Specifically, for every XP-generated pose, the distance between the ligand’s COM and the COM of the two Ni^2+^ ions were computed. This metric captures the likelihood of forming stable interactions within the catalytic pocket, under the assumption, supported by previous biochemical studies, that effective inhibitors typically position key functional groups in close proximity to the metal-coordination environment [73].

To enhance the practical relevance of the selected candidates, the acquisition cost of each ligand was evaluated as an additional prioritization factor. Commercial prices (USD per mg) were retrieved from major chemical suppliers (accessed on 14 November 2025) and consolidated into a single averaged estimate per compound:

- MedChem Express (https://www.medchemexpress.com);
- SelleckChem (https://www.selleckchem.com);
- TargetMol (https://www.targetmol.com);
- AdooQ Bioscience (https://www.adooq.com);
- MedKoo Bioscience (https://www.medkoo.com);
- Apollo Scientific (https://www.apolloscientific.co.uk);
- Aladdin Scientific (https://www.aladdinsci.com).

Rather than applying a strict cost threshold, this information was used to generate a comparative pricing profile across the entire candidate set. The final prioritization integrated three attributes simultaneously: (i) the most favorable XP docking energy observed within the ensemble, (ii) the shortest geometric distance between the ligand and the Ni^2+^ catalytic center, and (iii) the lowest average price per milligram. Compounds performing well across all three dimensions were considered the most viable for downstream validation, ensuring not only strong predicted affinity and appropriate catalytic positioning but also realistic feasibility for laboratory-scale acquisition.

### 3.6. Molecular Dynamics Simulations and MM-GBSA Binding Free Energy Estimation

All protein–ligand complexes that passed the XP refinement, catalytic-distance evaluation, and cost-based prioritization were subjected to all-atom MD simulations to assess the stability, conformational behavior, and persistence of key interactions within the *Hp*U catalytic pocket. MD simulations were performed using Desmond (Schrödinger Suite 2023-4) with the OPLS3e force field. In addition to the candidate ligands, three experimentally resolved *Hp*U–inhibitor complexes were included as controls [6,7]: PDB ID: 6ZJA containing 2-{[1-(3,5-dimethylphenyl)-1H-imidazol-2-yl]sulfanyl}-N-hydroxyacetamide (DJM), PDB ID: 6QSU containing β-mercaptoethanol (BME), and PDB ID: 1E9Y containing acetohydroxamic acid (HAE). These co-resolved ligands were retained during MD simulations to preserve the structural integrity of the catalytic site. Previous observations indicate that removal of bound ligands in *Hp*U leads to significant conformational rearrangements, including partial collapse and closure of the active site driven by flap-loop dynamics [16]. Such conformational states are not representative of the catalytically competent form required for ligand binding. Therefore, maintaining experimentally resolved ligands ensures sampling of functionally relevant open conformations of the active site, enabling meaningful comparison between candidate ligands and reference inhibitors under consistent structural conditions.

To avoid abrupt conformational drift of the docked poses and to allow a smooth structural adaptation of the active site, the MD simulations were initiated under a progressive restraint scheme applied to the ligand heavy atoms. During the first segment of the production trajectory (20 ns), a positional restraint of 10 kcal·mol^−1^·Å^−2^ was maintained to preserve the initial binding geometry. This force constant was then reduced stepwise as the simulation advanced: 5 kcal·mol^−1^·Å^−2^ for the next 30 ns, followed by 2 kcal·mol^−1^·Å^−2^ for an additional 30 ns. The restraints were completely lifted during the final 20 ns, allowing the system to evolve freely once the complex had reached a stable, equilibrated configuration. This staged relaxation minimizes artifacts associated with immediate ligand release while still permitting the ligand and neighboring residues to explore energetically favorable adjustments within the catalytic pocket. Throughout all simulations, the binuclear Ni^2+^ center was explicitly retained, and its coordination sphere was preserved according to the reference structural model. This ensured that the metal geometry, critical for urease catalysis and inhibitor binding, remained chemically consistent, preventing distortions that could otherwise arise from unconstrained MD sampling.

Only the unrestrained portion of each trajectory was used for downstream energetic and structural analyses. These analyses included the ligand RMSD, used to compare the positional stability of each candidate within the catalytic pocket relative to the three experimental controls. To obtain dynamic estimates of binding affinity, MM-GBSA calculations were performed using Prime MM-GBSA (Schrödinger Release 2023-3) with the VSGB 2.1 solvation model. For each system, snapshots were extracted exclusively from the fully unrestrained region of the simulation, ensuring that the resulting free-energy estimates reflected genuine dynamical behavior rather than restraint-induced conformations.

Binding free energies were computed according to:

$${∆G}_{bind}=\;G_{complex}-\left(G_{protein}+G_{ligand}\right)$$

With decomposition into *van der Waals*, Coulombic, lipophilic, hydrogen-bonding, solvation, and surface-area components. The resulting ${∆G}_{bind}$ values allowed a direct quantitative ranking of candidate ligands, complementing the SBVS metrics and enabling comparison with the three reference inhibitors.

### 3.7. Chemical Space Comparison Between Repurposed Candidates and Reported Urease Inhibitors

To contextualize the structural novelty of the selected repurposed candidates, a comparative chemical space analysis was performed against previously reported urease inhibitors compiled from public bioactivity repositories (UI-ref). A curated reference dataset of UI-ref was constructed by integrating data from three major public repositories: ChEMBL [74], BindingDB [75], and PubChem BioAssay [76]. These databases provide manually curated compounds with experimentally validated bioactivity data extracted from the literature.

Data retrieval was performed on 30 November 2025, using the following criteria:

- ChEMBL: A text-based search using the term “urease” was conducted. Results were restricted to protein-type targets (single protein or protein family) and functional assays. Only compounds with quantitative bioactivity measurements (IC_50_, K_i_, or EC_50_) were retained.
- BindingDB: The term “urease” was used as a query, and results were filtered to include only compounds with experimentally determined binding data and associated quantitative activity values.
- PubChem: A search using “urease inhibitor” was conducted, limiting retrieval to compounds classified as active in experimental assays, with preference given to confirmatory assay results.

The retrieved records were merged into a unified UI-ref dataset. Each entry included the following metadata:

- Compound identifier;
- Canonical SMILES;
- Source database (ChEMBL, BindingDB, or PubChem);
- Target organism (when available);
- Type of bioactivity measurement (IC_50_, K_i_, EC_50_);
- Activity value normalized to molar units (M);
- Standardized InChIKey retained as a unique structural identifier.

Duplicate structures were resolved using InChIKey matching to ensure structural uniqueness across databases.

To quantify structural relationships between the nine repurposed candidates and the UI-ref dataset, a fingerprint-based chemical space analysis was performed. All molecules were encoded using ECFP4 circular fingerprints (Morgan fingerprints, radius = 2) with fixed-length binary vectors. Structural similarity was quantified using the Tanimoto coefficient, defined as:

$$T\left(A,B\right)=\;\frac{c}{a+b-c}$$

where *a* and *b* are the number of on-bits in fingerprints A and B, respectively, and *c* is the number of shared on-bits.

A bidirectional nearest-neighbor (NN) approach was implemented to assess structural overlap between the candidate set and the UI-Ref dataset:

1. Candidates → UI-ref: For each of the nine repurposed candidates, the most similar molecule in UI-ref was identified, and its Tanimoto similarity value was recorded.
2. UI-ref → Candidates: For each molecule in UI-ref (n = 338), the most similar candidate compound was identified.

This bidirectional strategy ensures evaluation of both: how close each candidate is to known inhibitors and how well the candidate set covers the chemical space of reported UI-ref.

Similarity distributions were summarized using median, 10th percentile (p10), and 90th percentile (p90) statistics. Conventional similarity thresholds were considered for contextual interpretation:

- T ≥ 0.85: Highly similar (near analog);
- T ≥ 0.70: Structurally close analog;
- T ≥ 0.50: Moderate scaffold similarity.

The absence of values ≥ 0.50 was interpreted as evidence of limited scaffold overlap between chemical spaces. The resulting similarity distributions were visualized using violin plots with superimposed data points, enabling comparative assessment of structural proximity in both directions.

### 3.8. Well-Tempered Metadynamics (WT-MetaD)

*WT-MetaD* simulations were performed to characterize the free-energy landscape associated with ligand retention inside the *Hp*U catalytic cavity and to evaluate the likelihood of spontaneous dissociation from the metal-centered binding region. The simulations followed the same protocol previously validated by our group for *Hp*U inhibitors (Ríos-Rozas et al., 2025 [10]), where the choice of collective variables, biasing parameters, and convergence criteria was optimized to capture the slow conformational transitions governing urease–ligand stability.

Two collective variables (CVs) were selected based on their ability to capture the essential degrees of freedom governing inhibitor retention:

- CV1, the distance between the COM of the ligand heavy atoms and the binuclear Ni^2+^ center;
- CV2, the distance between the ligand COM and the COM of the surrounding catalytic residues (KCX219, H274, C321, D362, A365).

These CVs correspond to the same geometric descriptors shown in our previous work to distinguish strongly bound inhibitors from weak or transient binders. For each ligand, three independent WT-MetaD replicas of 100 ns were generated using the equilibrated structures obtained from the unrestricted MD phase. The same metadynamics parameters reported in the previous study were employed without modification, ensuring methodological consistency and enabling direct comparison with experimentally validated controls.

Free-energy surfaces (FES) and associated probability density maps were derived from the reconstructed bias potentials, allowing identification of bound and partially dissociated states. These analyses were used to quantify the depth and stability of the ligand’s free-energy basin relative to the catalytic Ni^2+^ center and to benchmark each candidate against the three controls. In this study, WT-MetaD served as the final refinement step to prioritize compounds that not only display favorable docking and MM-GBSA energies but also exhibit energetically persistent binding modes under enhanced sampling conditions.

## 4. Conclusions

In this work, we developed and validated a stringent, multi-layered computational pipeline for the identification of novel urease inhibitors through drug repurposing, explicitly adapted to the structural and physicochemical constraints of *Hp*. By integrating multiparametric filtering, ED, high-precision refinement, MD simulations, and enhanced sampling, we demonstrate that it is possible to reliably prioritize candidate molecules that are not only energetically favorable but also structurally stable and chemically distinct from previously reported UI.

A key outcome of this study is the identification of a reduced set of high-confidence candidates that consistently outperform reference inhibitors across multiple independent metrics, including docking, MM-GBSA, and WT-MetaD free-energy landscapes. Importantly, the absence of significant similarity to known UI scaffolds indicates that this workflow enables the exploration of previously uncharacterized chemical space, rather than incremental optimization of existing chemotypes. This represents a relevant advance in the context of urease-targeted drug discovery, where structural diversity remains limited.

From a translational perspective, the fact that all prioritized compounds possess known pharmacokinetic and safety profiles significantly lowers the barrier for experimental validation. Based on our results, we propose a clear validation pathway: (i) initial in vitro urease inhibition assays to determine IC_50_ values and confirm activity against the Ni^2+^-dependent catalytic site, (ii) metal-dependence and reversibility studies to elucidate the mechanism of inhibition, and (iii) cellular assays in *Hp* models to evaluate anti-virulence effects, particularly under acidic conditions that mimic the gastric environment. Compounds showing consistent activity across these stages could be rapidly advanced toward in vivo validation or combination therapies with existing antibiotics.

Beyond the specific case of urease inhibition, this study highlights the importance of combining receptor flexibility, rigorous energetic evaluation, and chemical space analysis within a unified framework. The consistent performance of ensemble-based approaches and free-energy methods underscores their value in reducing false positives and improving prioritization reliability in virtual screening campaigns.

Overall, this work establishes a practical and transferable strategy for drug repurposing against metalloenzymes and challenging bacterial targets. By bridging computational prediction with experimentally actionable outputs, the proposed pipeline provides a robust foundation for accelerating the identification of novel anti-virulence agents. Future studies should focus on experimental validation of the proposed candidates and on the integration of adaptive or machine learning-based scoring schemes to further refine prioritization and expand the accessible chemical space.

## Figures and Tables

**Table 1:** WT-MetaD–derived free energy barriers for ligand dissociation. Free energy differences (ΔF, kcal/mol) extracted from WT-MetaD simulations for each ligand along two independent collective variables (CV1 and CV2). ΔF values were calculated as the difference between the maximum and minimum free energy sampled along each CV. The mean (ΔF) corresponds to the arithmetic average of ΔFCV1 and ΔFCV2 and is used as an overall metric to rank ligand binding stability. Candidate compounds are reported together with the reference control inhibitors (DJM, BME, and HAE) in bold.

Molecule	${∆F}^{CV1}$	${∆F}^{CV2}$	$Mean\left(∆F\right)$
ASCORBIC-ACID	8.601	6.081	7.341
FOSFOSAL	6.613	9.082	7.847
FENBUFEN	11.370	7.514	9.442
OXAPROZIN	12.781	7.114	9.947
**DJM**	**13.030**	**10.266**	**11.648**
TILUDRONATE	14.328	11.104	12.716
MINOXIDIL	14.275	11.463	12.869
AMFENAC	13.263	12.567	12.915
GABPENTIN	15.288	13.657	14.473
TRANEXAMIC-ACID	16.364	14.208	15.286
**BME**	**18.056**	**12.524**	**15.290**
**HAE**	**22.694**	**16.740**	**19.717**

## Data Availability

Data supporting the findings of this study are available within the electronic Supporting Information. The scripts and data were deposited in GitHub link: https://github.com/DanielBustosG/Drug-Repurposing-Uncovers-New-Chemical-Scaffolds-as-Potent-Urease-Inhibitors.git (accessed on 12 March 2026).

## Supplementary Materials

The following supporting information can be downloaded at: https://www.mdpi.com/article/10.3390/ijms27083561/s1.

## Abbreviations

Hp	*Helicobacter pylori*
HpU	*Helicobacter pylori* urease
UI	Urease inhibitors
UI-ref	Reference urease inhibitors dataset
SBVS	Structure-Based Virtual Screening
MPS	Multiparametric Scoring
ED	Ensemble Docking
MD	Molecular Dynamics
MM-GBSA	Molecular Mechanics Generalized Born Surface Area
WT-MetaD	Well-Tempered Metadynamics
CV	Collective Variable
COM	Center of Mass
XP	Extra Precision
RMSD	Root Mean Square Deviation
RMSF	Root Mean Square Fluctuation
FES	Free Energy Surface
ΔF	Free energy difference
tPSA	Topological Polar Surface Area
RB	Rotatable Bonds
PBF	Plane of Best Fit
ECFP4	Extended-Connectivity Fingerprint (radius 2)
