# Supplementary material for: Drug Repurposing Uncovers New Chemical Scaffolds as Potent Urease Inhibitors: A Comprehensive Computational Study
Source: Int J Mol Sci. 2026 Apr 16;27(8):3561. doi: 10.3390/ijms27083561 (PMC13115936; doi:10.3390/ijms27083561)
Supplement: Supplementary file 1 [file ijms-27-03561-s001.zip › ijms-4228517-supplementary.pdf]

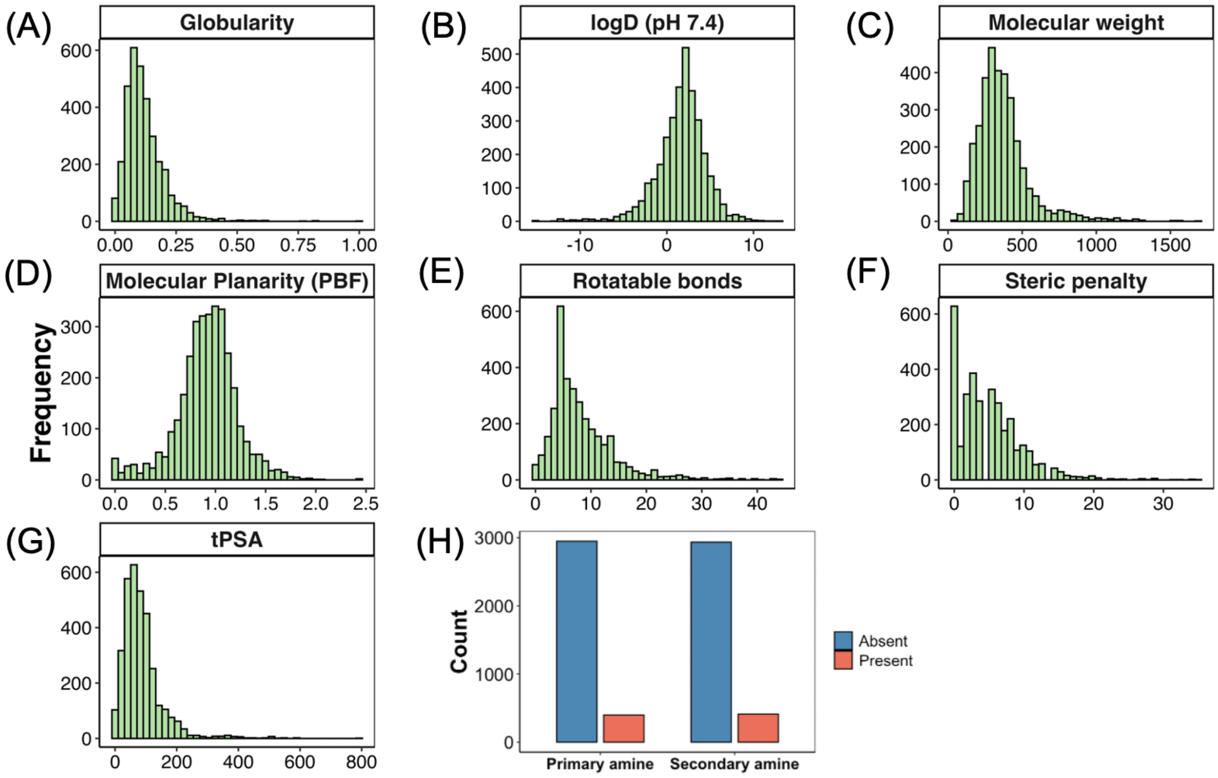

Figure S1. Distribution of physicochemical descriptors and amine frequencies in the repurposing dataset. (A–G) Histograms showing the distribution of seven physicochemical descriptors considered in the MPS function: (A) globularity, (B) logD at pH 7.4, (C) molecular weight, (D) molecular planarity (PBF), (E) rotatable bonds, (F) steric penalty, and (G) topological polar surface area (tPSA). These properties were selected based on permeability-determining features reported by Richter et al. and subsequent studies on antibiotic chemical space. Each panel displays the full range of values across the curated repurposing library, illustrating the intrinsic chemical diversity of the dataset prior to MPS-based refinement. (H) Bar plots summarizing the prevalence of primary and secondary amines, shown as the number of molecules containing (Present) or lacking (Absent) these functionalities.

Table S1. Descriptive statistics of XP docking scores and ligand–Ni^{2+} distances used for candidate prioritization. Summary statistics of XP Glide docking scores (kcal/mol) and average ligand–Ni^{2+} distances (Å) for compounds evaluated in the XP refinement stage. Statistics are reported for three datasets: (i) the global set including the pose #1 for each complex across frames, (ii) the selected subset comprising complexes within the joint energy–distance thresholds defined in Fig. 4A (DockingScore ≤ −6.805 kcal/mol and Average Distance ≤ 2.094 Å), and (iii) the selected & unique dataset, where a single representative pose/frame per ligand was retained by selecting the lowest XP docking score. Q1 and Q3 correspond to the first and third quartiles, respectively. S.D.: standard deviation.

Criterium	Dataset	# Ligands	Q1	Median	Mean	Q3	Minimum	Maximum	S.D.
Energy (kcal/mol)	Global	2717	-6.805	-6.040	-5.767	-4.447	-12.432	3.486	1.749
	Selected	277	-7.974	-7.388	-7.702	-7.032	-11.264	-6.805	0.958
	Selected & Unique	55	-8.310	-7.806	-7.984	-7.275	-11.264	-6.848	0.912
Distance (Å)	Global	2717	2.094	2.143	2.392	2.395	1.907	7.817	0.657
	Selected	277	1.996	2.034	2.026	2.052	1.907	2.093	0.036
	Selected & Unique	55	1.996	2.031	2.024	2.048	1.971	2.092	0.032

Table S2. Physicochemical, docking, distance, and commercial availability descriptors of the selected urease inhibitor candidates. Summary of the nine candidate compounds selected after ensemble docking, XP rescoring, metal–distance filtering, and price-based prioritization. For each ligand, the table reports the average commercial price (Price_Mean, USD) and its standard deviation (Price_SD) across the evaluated distributors, the XP docking score (DockingScore, kcal/mol), the average distance to the catalytic Ni^{2+} ions (Average Distance, Å) and its standard deviation (SD Distance, Å), the number of vendors in which the compound was available (out of seven evaluated), and the molecular weight (MW, g/mol). Two-dimensional chemical structures of the selected compounds are also shown. This multiparametric selection integrates energetic, geometric, physicochemical, and feasibility criteria and defines the final set of ligands advanced to molecular dynamics simulations and enhanced sampling analyses. Table ordered by XP docking score.

Molecule	Structure	XP docking score (kcal/mol)	Average Ni–ligand distance (Å)	Ni–ligand distance SD (Å)	Average price (USD)	Price SD (USD)	Available vendors (n/7)	Molecular weight (g/mol)
MINOXIDIL	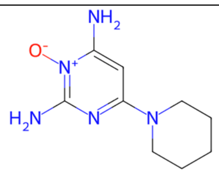	-11.264	2.047	0.125	0.580	1.376	7/7	209.248
GABAPENTIN	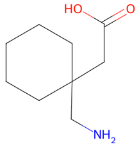	-10.367	2.034	0.046	2.415	2.724	6/7	171.237
TILUDRONATE	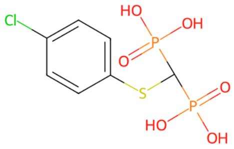	-10.252	2.024	0.066	23.436	16.725	5/7	318.608
OXAPROZIN	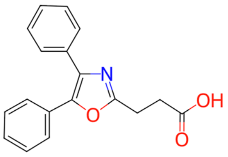	-9.255	2.036	0.043	1.824	1.796	6/7	293.317
FOSFOSAL	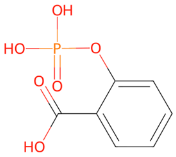	-9.181	2.067	0.100	9.480	9.058	6/7	218.100
TRANEXAMIC-ACID	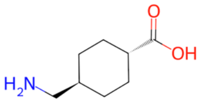	-9.153	1.975	0.069	0.650	1.495	6/7	157.210
FENBUFEN	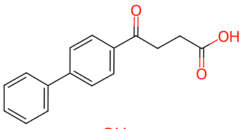	-9.055	2.079	0.008	0.296	0.419	7/7	254.281
ASCORBIC-ACID	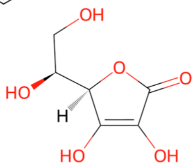	-8.806	2.078	0.020	0.552	1.388	7/7	176.124
AMFENAC	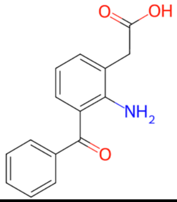	-8.801	1.988	0.155	37.540	81.878	5/7	255.273

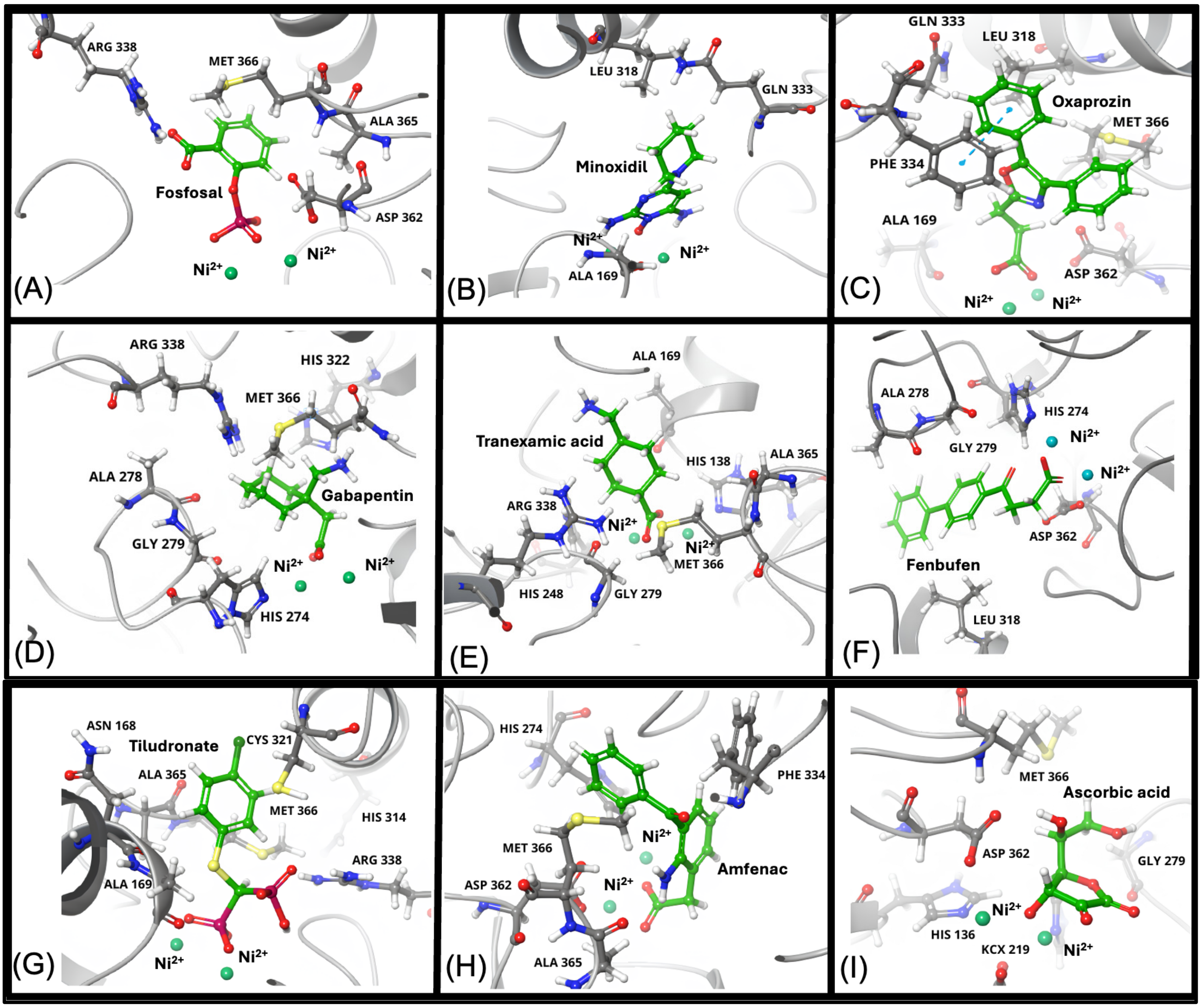

Figure S2. Representative docking poses of the nine selected urease inhibitor candidates within the *Hp*U catalytic site: A) Fosfosal, B) Minoxidil, C) Oxaprozin, D) Gabapentin, E) Tranexamic acid, F) Fenbufen, G) Tiludronate, H) Amfenac, and I) Ascorbic acid. The structures correspond to the selected XP docking poses that satisfied both energetic (XP ≤ −6.805 kcal/mol) and geometric (Ni^{2+} proximity) criteria and were subsequently used as starting configurations for molecular dynamics simulations. Ligands are shown in stick representation, while the catalytic Ni^{2+} ions are represented as green spheres. The figure highlights the spatial orientation of each compound relative to the bimetallic center, illustrating their potential to engage the catalytic site through coordination, hydrogen bonding, and steric complementarity.

Table S3. Descriptive statistics of protein backbone RMSD during molecular dynamics simulations. Protein backbone RMSD (Å) calculated for each protein–ligand complex over the last 20 ns of unrestrained molecular dynamics simulations. For each system, minimum, first quartile (Q1), median, mean, third quartile (Q3), maximum, and standard deviation (S.D.) values are reported. The table summarizes the conformational stability of the protein backbone as modulated by binding of each candidate compound and the reference control inhibitors (DJM, HAE, and BME). Table ordered by median.

Molecule	Minimum	Q1	Median	Mean	Q3	Maximum	S.D.
TRANEXAMIC-ACID	0.951	1.105	1.180	1.192	1.250	1.524	0.117
FENBUFEN	0.857	1.090	1.241	1.272	1.429	1.917	0.241
OXAPROZIN	1.072	1.196	1.273	1.304	1.400	1.747	0.149
HAE	1.034	1.263	1.319	1.333	1.384	1.763	0.155
DJM	1.077	1.184	1.353	1.362	1.510	1.763	0.196
MINOXIDIL	1.038	1.279	1.377	1.414	1.554	2.127	0.214
BME	1.041	1.303	1.436	1.399	1.493	1.644	0.153
AMFENAC	1.051	1.360	1.488	1.500	1.633	1.849	0.186
GABAPENTIN	1.092	1.382	1.490	1.485	1.585	1.937	0.197
ASCORBIC-ACID	0.929	1.406	1.517	1.516	1.668	2.054	0.238
FOSFOSAL	1.161	1.458	1.522	1.535	1.614	1.869	0.158
TILUDRONATE	1.143	1.464	1.590	1.569	1.679	1.959	0.192

Table S4. **Descriptive statistics of ligand RMSD during molecular dynamics simulations**. Ligand RMSD (Å) computed relative to the protein for each ligand during the final 20 ns of unrestrained molecular dynamics simulations. Minimum, first quartile (Q1), median, mean, third quartile (Q3), maximum, and standard deviation (S.D.) values are reported for all candidate compounds and reference control inhibitors (DJM, HAE, and BME). Table ordered by median.

Molecule	Minimum	Q1	Median	Mean	Q3	Maximum	S.D.
MINOXIDIL	0.276	0.362	0.566	0.571	0.735	1.192	0.228
HAE	0.458	0.674	0.783	0.801	0.954	1.178	0.191
TILUDRONATE	0.312	0.739	0.836	0.893	1.027	1.758	0.299
BME	0.356	0.687	0.858	0.919	1.041	2.013	0.353
FOSFOSAL	0.275	0.669	0.899	0.874	1.083	1.559	0.281
OXAPROZIN	0.511	0.821	0.964	0.961	1.056	1.577	0.236
TRANEXAMIC-ACID	0.354	0.676	1.053	1.044	1.314	2.026	0.462
FENBUFEN	0.565	0.975	1.131	1.291	1.315	3.154	0.592
AMFENAC	0.765	1.015	1.143	1.192	1.350	2.016	0.262
ASCORBIC-ACID	0.751	1.006	1.227	1.211	1.411	1.688	0.238
GABAPENTIN	0.484	1.068	1.280	1.402	1.713	2.774	0.495
DJM	1.092	1.751	2.455	2.409	2.918	3.971	0.782

Table S5. Descriptive statistics of binding free energy through MM-GBSA during the final 20 ns of unrestrained molecular dynamics simulations. Descriptive statistics of protein-ligand binding free energy (MM-GBSA, kcal/mol) computed during the final 20 ns of unrestrained molecular dynamics simulations. Minimum, first quartile (Q1), median, mean, third quartile (Q3), maximum, and standard deviation (S.D.) values are reported for all candidate compounds and reference control inhibitors (DJM, HAE, and BME). Table ordered by median.

Molecule	Minimum	Q1	Median	Mean	Q3	Maximum	S.D.
TILUDRONATE	-87.010	-78.056	-75.999	-76.594	-73.967	-71.716	3.658
FENBUFEN	-81.742	-77.691	-75.735	-75.563	-74.076	-65.503	3.189
OXAPROZIN	-77.002	-72.046	-70.158	-69.430	-67.136	-62.561	3.681
FOSFOSAL	-78.498	-65.023	-61.581	-62.055	-59.606	-50.758	4.742
AMFENAC	-70.067	-63.815	-61.239	-61.734	-59.745	-54.577	3.881
TRANEXAMIC-ACID	-52.009	-48.316	-47.037	-46.898	-45.280	-41.893	2.275
MINOXIDIL	-45.289	-38.180	-36.537	-36.579	-34.472	-31.522	2.871
ASCORBIC-ACID	-42.574	-38.534	-35.702	-35.917	-33.759	-21.163	4.066
GABAPENTIN	-41.590	-35.024	-32.677	-32.881	-30.953	-26.407	3.139
DJM	-30.619	-23.450	-18.990	-18.926	-14.684	-4.275	6.845
HAE	-12.673	-10.810	-9.381	-9.248	-7.928	-3.637	1.980
BME	-1.028	8.131	10.939	10.863	13.633	23.138	4.783

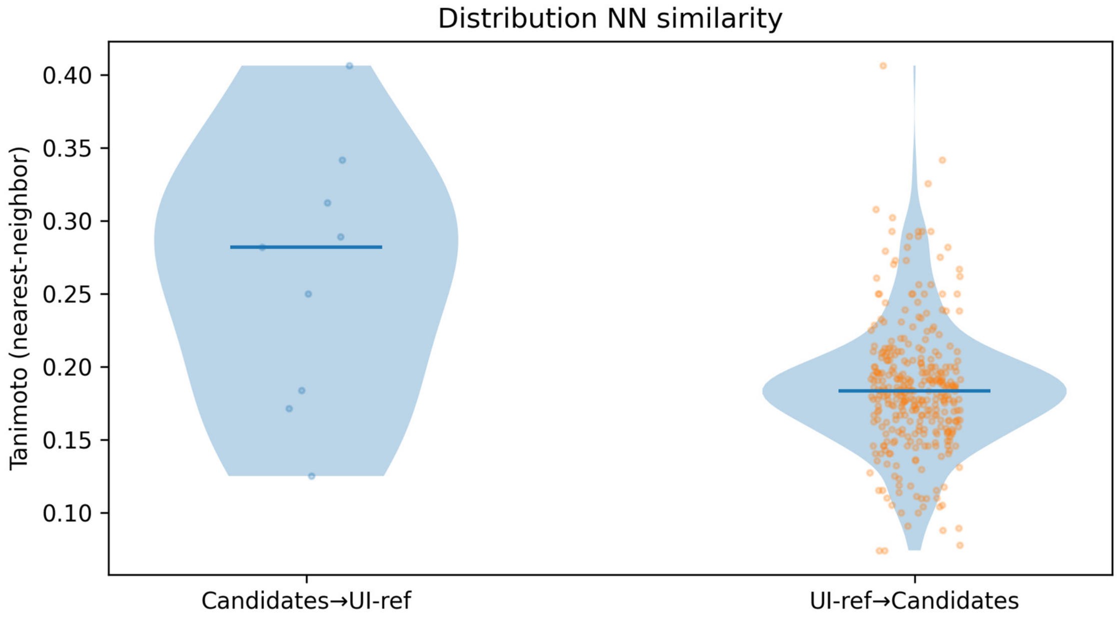

Figure S3. Bidirectional nearest-neighbor (NN) similarity between repurposed candidates and reported urease inhibitors (UI-ref). Violin plots show the distribution of maximum (nearest-neighbor) Tanimoto similarities computed from ECFP4 (Morgan radius = 2) fingerprints in both directions: Candidates→UI-ref (for each candidate, similarity to its most similar UI-ref compound; n = 9) and UI-ref→Candidates (for each UI-ref compound, similarity to its most similar candidate; n = 338). Horizontal bars indicate the median. The low similarity values in both directions (Candidates→ UI-ref median = 0.282; p10–p90 = 0.162–0.354; UI-ref→Candidates median = 0.183; p10–p90 = 0.138–0.238) and the absence of pairs with NN ≥ 0.5 support limited chemical-space overlap between the candidate set and previously reported urease inhibitors, consistent with structural novelty under conventional fingerprint-based similarity criteria.

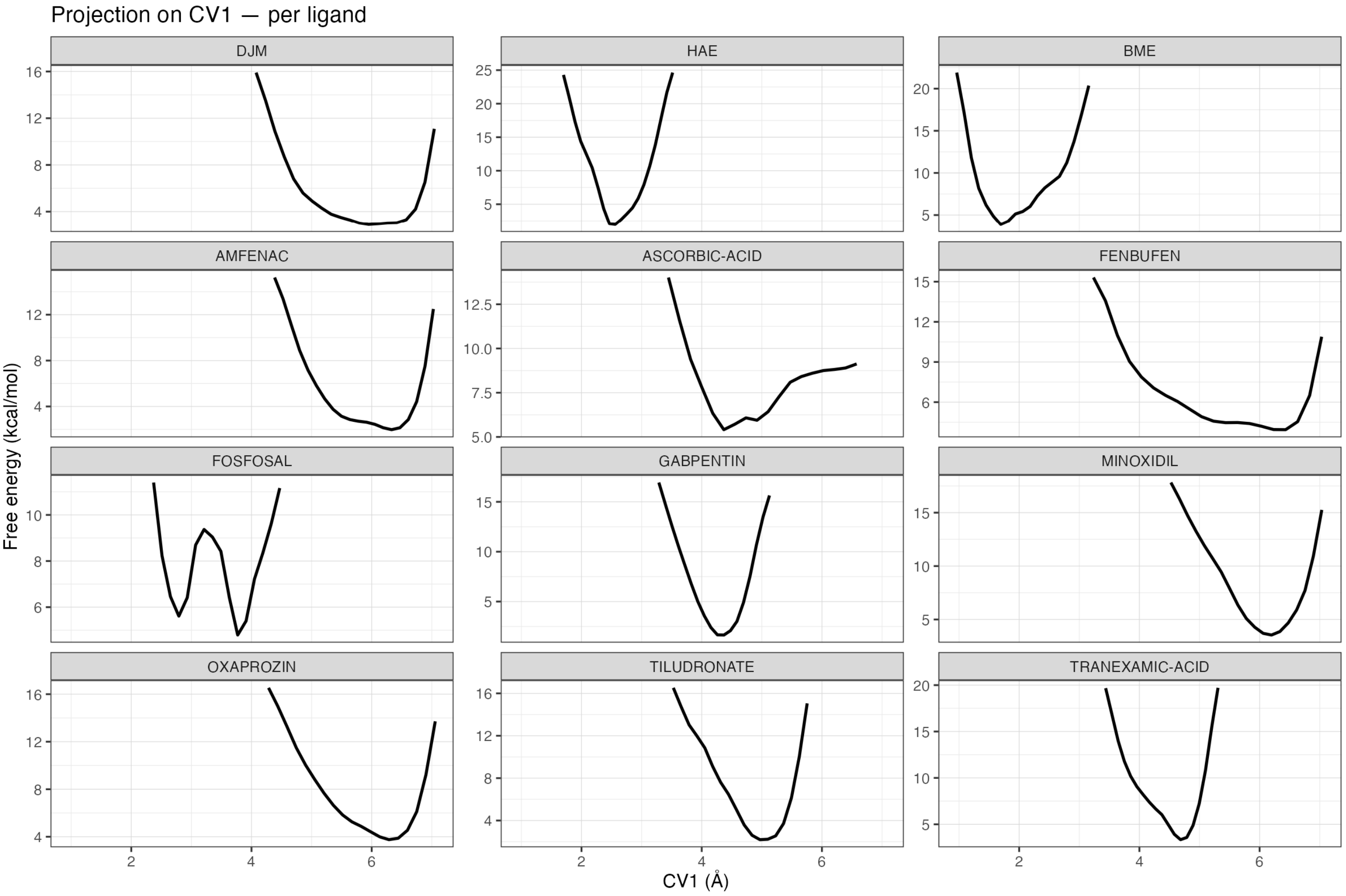

Figure S4. One-dimensional free-energy projections along CV1 for individual ligands. One-dimensional free-energy profiles obtained by projection of the WT-MetD free-energy surfaces onto collective variable CV1 for each candidate ligand and reference control (DJM, HAE, and BME). CV1 describes the distance between the center of mass (COM) of ligand heavy atoms and the bimetallic Ni^{2+} cluster of the urease active site. The resulting free-energy profiles report on the thermodynamic cost associated with ligand displacement relative to the metal center, providing a direct measure of metal-site proximity and binding persistence. Ligands exhibiting narrow, deep minima along CV1 indicate stable coordination near the catalytic core, whereas broader or shifted minima reflect increased positional variability. These projections reveal the curvature and confinement of the dominant basin, which directly determine the ΔF amplitude reported in Table 1.

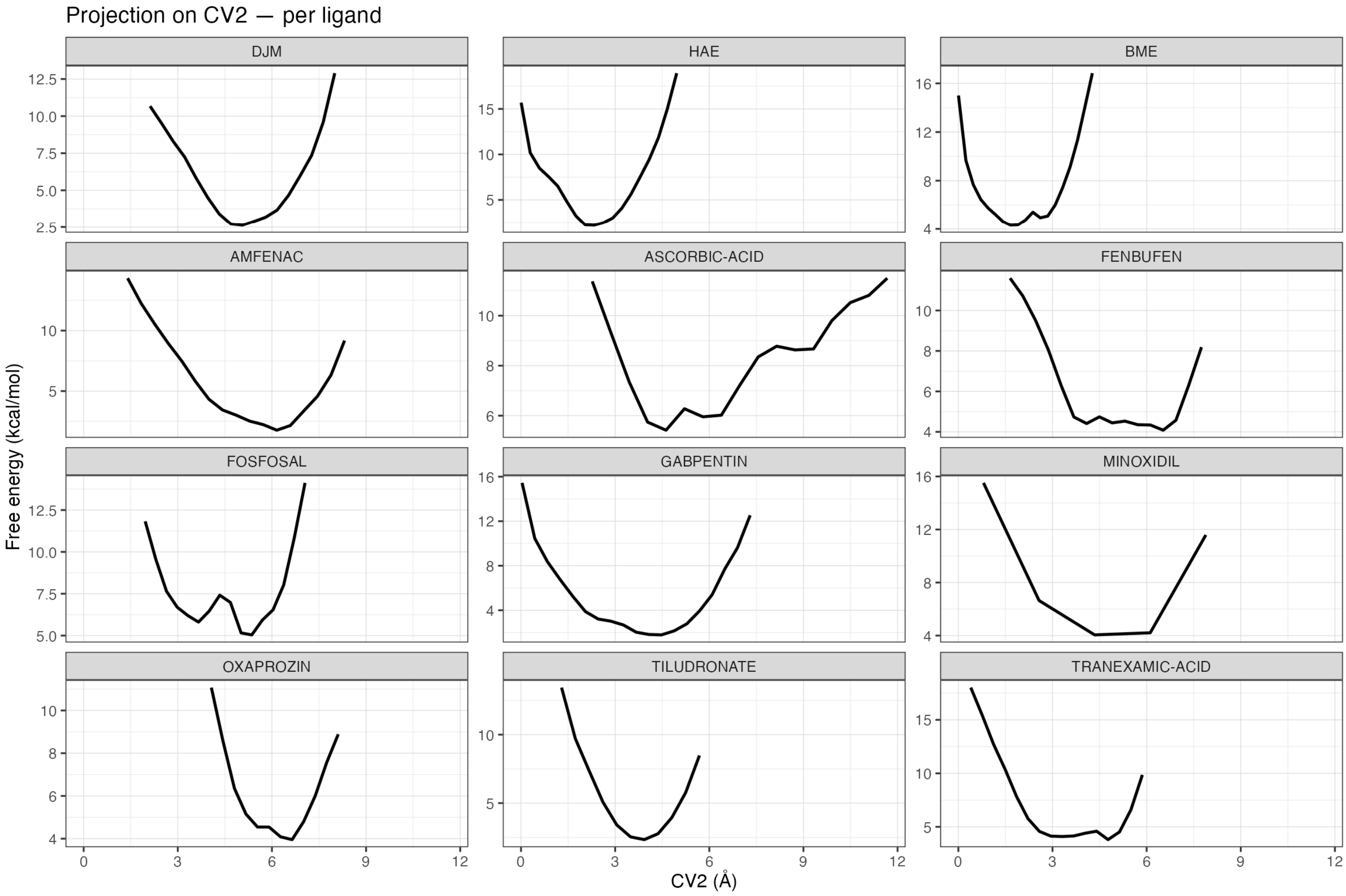

Figure S5. One-dimensional free-energy projections along CV2 for individual ligands. One-dimensional free-energy profiles obtained by projection of the WT-MetD free-energy surfaces onto collective variable CV2 for each candidate ligand and reference control (DJM, HAE, and BME). CV2 corresponds to the distance between the center of mass (COM) of the ligand and the COM of key catalytic residues (KCX219, H274, C321, D362, and A365) forming the urease active-site environment. The free-energy curves represent the minimum free-energy pathway along CV2, highlighting the location, depth, and curvature of the dominant thermodynamic basins. Differences in basin width and steepness reflect ligand-specific conformational flexibility and the strength of confinement within the catalytic pocket, complementing the two-dimensional free-energy landscapes shown in Fig. 6. These projections reveal the curvature and confinement of the dominant basin, which directly determine the ΔF amplitude reported in Table 1.
